# Characterization of a lytic *Escherichia coli* phage CE1 and its potential use in therapy against avian pathogenic *Escherichia coli* infections

**DOI:** 10.3389/fmicb.2023.1091442

**Published:** 2023-02-16

**Authors:** Zhaohui Tang, Ning Tang, Xinwei Wang, Huiying Ren, Can Zhang, Ling Zou, Lei Han, Longzong Guo, Wenhua Liu

**Affiliations:** ^1^College of Veterinary medicine, Qingdao Agricultural University, Qingdao, Shandong, China; ^2^College of Chemistry and Pharmaceutical Science, Qingdao Agricultural University, Qingdao, Shandong, China; ^3^Shandong Yisheng Livestock & Poultry Breeding Co., Ltd., Yantai, Shandong, China

**Keywords:** phage vB_EcoM_CE1, genome sequencing, broiler, avian pathogenic *Escherichia coli*, bactericidal activity, phage therapy

## Abstract

The high incidence of Avian pathogenic *Escherichia coli* (APEC) in poultry has resulted in significant economic losses. It has become necessary to find alternatives to antibiotics due to the alarming rise in antibiotic resistance. Phage therapy has shown promising results in numerous studies. In the current study, a lytic phage vB_EcoM_CE1 (short for CE1) against *Escherichia coli* (*E. coli*) was isolated from broiler feces, showing a relatively wide host range and lysing 56.9% (33/58) of high pathogenic strains of APEC. According to morphological observations and phylogenetic analysis, phage CE1 belongs to the *Tequatrovirus* genus, *Straboviridae* family, containing an icosahedral capsid (80 ~ 100 nm in diameter) and a retractable tail (120 nm in length). This phage was stable below 60°C for 1 h over the pH range of 4 to 10. Whole-genome sequencing revealed that phage CE1 contained a linear double-stranded DNA genome spanning 167,955 bp with a GC content of 35.4%. A total of 271 ORFs and 8 tRNAs were identified. There was no evidence of virulence genes, drug-resistance genes, or lysogeny genes in the genome. The *in vitro* test showed high bactericidal activity of phage CE1 against *E. coli* at a wide range of MOIs, and good air and water disinfectant properties. Phage CE1 showed perfect protection against broilers challenged with APEC strain *in vivo*. This study provides some basic information for further research into treating colibacillosis, or killing *E. coli* in breeding environments.

## 1. Introduction

*Escherichia coli* (*E. coli*), an important member of the *Enterobacteriaceae* family, is widely distributed in various ecosystems. It plays a vital role in maintaining the homeostasis of the host as a normal components of human and animal gut microbiota (Holmes et al., 2011; Deepak Kumar, 2022). However, humans and animals can be infected with some virulent serotypes of *E. coli* that cause colibacillosis, a notorious disease (Juliana et al., 2020; Wilczynski et al., 2022). The avian pathogenic *Escherichia coli* (APEC) is one of these pathogens that causes morbidity and mortality in chickens worldwide with symptoms of airsacculitis, perihepatitis, pericarditis, peritonitis and arthritis (Apostolakos et al., 2021; Kim et al., 2020). The growing number of multidrug-resistant strains poses a serious therapeutic challenge for APEC (Azam et al., 2020; Montoro-Dasi et al., 2020). In view of many drawbacks associated with antibiotics, the European Union has promulgated a series of regulations prohibiting the use of antibiotics as growth promoters and reducing the use of certain antibiotics in animal husbandry, and farmers are willing to reduce antibiotics if effective alternatives are available (Millet et al., 2011; Huyghebaert et al., 2011). In China, according to the National Action Plan for Reducing the Use of Veterinary Antibiotics (2021–2025) issued by the Ministry of Agriculture and Rural Affairs of People’s Republic of China, antibiotic use is strictly regulated (http://www.gov.cn/zhengce/zhengceku/2021-10/25/content_5644815.htm). As a result, innovative techniques are increasingly being applied against conventional antibiotics. Several antibacterial agents have been studied in recent years, including antimicrobial peptides, essential oils, probiotics, and bacteriophages (phages) (Wu et al., 2020; Jiang et al., 2021; Rinaldi et al., 2021).

Phages are among the most abundant and diverse viruses in the biosphere, with a specific and efficient bactericidal effect. Consequently, phages can be used as alternatives or synergized antibiotics (Rehman et al., 2019; Hampton et al., 2020). Scientific evidence pointing to the benefits of phage therapy on human and animal has steadily developed in recent decades (Hon et al., 2022; Goode et al., 2003; Atterbury et al., 2007; Denou et al., 2009). Additionally, it had been reported to be used as indicators to monitor the safety of water (Maganha et al., 2021). As the number of available phages is still relatively limited, it is urgent to isolate safe, highly lytic, and well characterized phages for phage therapy.

In this study, a newly isolated virulent phage against APEC strains was characterized, the whole-genome was sequenced, and its biological properties were characterized. In addition, bacteriostasis was evaluated *in vitro* and *in vivo*. All the experiments were designed to verify that phage CE1 was a promising candidate for control of *E. coli*.

## 2. Materials and methods

### 2.1. Animals

Healthy 1-day-old broilers (Hubbard) were offered by Shandong Yisheng Livestock & Poultry Breeding Co., Ltd., Shandong, China. The experiments in this study strictly followed the national guidelines for experimental animal welfare issued by the Ministry of Science and Technology of People’s Republic of China in 2006 (Guiding Opinions on Kindly Treating Laboratory Animals), and were approved by the Animal Welfare and Research Ethics Committee at Qingdao Agricultural University, Shandong, China (Approved No: 2021–98). Animals were treated humanly in the experiment, and every effort was made to reduce the suffering of the animals. Broiler groups were housed in separate rearing isolators equipped with wire mesh floors, two nipple drinkers, one feeder and one light. Room housing the isolators was air-conditioned and air was pumped into the isolators.

### 2.2. Samples and bacterial strains

Feces samples collected from broiler farms in Shandong, China were used to isolate phages. As shown in Tables 1, 58 highly pathogenic APEC strains used in this study were previously isolated from different origins (ovary of culled broiler, clinical dead broiler, 1-day-old broiler, and yolk of dead embryo) and stored in the Veterinary Microbiology Laboratory of Qingdao Agricultural University, Qingdao, Shandong, China. All strains were cultured at 37°C in Luria-Bertani (LB) broth, and stored in LB broth supplemented with 30% glycerol at −80°C. A high pathogenic APEC strain named SD-C888 (isolated from ovary of a culled broiler) was used as an indicator strain for phage isolation, purification, and propagation.

**Table 1:** Host range of phage CE1 against APEC strains.

No.	Strains	EOP	Mortality rate of embryo (%)	Source
1	*E. coli* 501	-	70	Clinical dead broiler, Yantai, China (2010)
2	*E. coli* 506	Medium	70	1-day-old broiler, Yantai, China (2010)
3	*E. coli* 507	High	80	Yolk of dead embryo, Yantai, China (2010)
4	*E. coli* 508	-	100	1-day-old broiler, Qingdao, China (2011)
5	*E. coli* 509	-	70	Clinical dead broiler, Weifang, China (2011)
6	*E. coli* 510	-	70	Clinical dead broiler, Yantai, China (2011)
7	*E. coli* 511	-	60	Clinical dead broiler, Yantai, China (2012)
8	*E. coli* 512	-	60	Clinical dead broiler, Qingdao, China (2012)
9	*E. coli* 513	low	70	Clinical dead broiler, Weifang, China (2012)
10	*E. coli* 514	-	90	Clinical dead broiler, Yantai, China (2013)
11	*E. coli* 515	-	70	Clinical dead broiler, Weifang, China (2013)
12	*E. coli* 516	low	70	Clinical dead broiler, Weifang, China (2014)
13	*E. coli* 517	High	50	Clinical dead broiler, Yantai, China (2014)
14	*E. coli* 518	Medium	70	1-day-old broiler, Yantai, China (2014)
15	*E. coli* 519	Medium	80	1-day-old broiler, Weifang, China (2014)
16	*E. coli* 520	High	60	1-day-old broiler, Qingdao, China (2014)
17	*E. coli* 540	low	80	Clinical dead broiler, Qingdao, China (2015)
18	*E. coli* 541	High	50	1-day-old broiler, Yantai, China (2015)
19	*E. coli* 547	-	50	1-day-old broiler, Weifang, China (2015)
20	*E. coli* 549	-	60	Yolk of dead embryo, Yantai, China (2015)
21	*E. coli* 552	low	70	Clinical dead broiler, Yantai, China (2015)
22	*E. coli* 554	High	80	Clinical dead broiler, Weifang, China (2015)
23	*E. coli* 555	-	50	1-day-old broiler, Qingdao, China (2016)
24	*E. coli* 557	low	80	Clinical dead broiler, Jimo, China (2015)
25	*E. coli* 559	-	50	1-day-old broiler, Weifang, China (2016)
26	*E. coli* 560	Medium	90	1-day-old broiler, Yantai, China (2016)
27	*E. coli* 564	Medium	50	1-day-old broiler, Weihai, China (2016)
28	*E. coli* 565	-	60	Yolk of dead embryo, Yantai, China (2016)
29	*E. coli* 569	Medium	80	Yolk of dead embryo, Qingdao, China (2016)
30	*E. coli* 570	low	80	Yolk of dead embryo, Yantai, China (2016)
31	*E. coli* 578	-	50	Ovary of culled broiler, Yantai, China (2016)
32	*E. coli* 612	low	70	Yolk of dead embryo, Jinan, China (2016)
33	*E. coli* 613	High	60	Yolk of dead embryo, Binzhou, China (2016)
34	*E. coli* 615	Medium	50	Clinical dead broiler, Yantai, China (2016)
35	*E. coli* 618	-	50	1-day-old broiler, Yantai, China (2017)
36	*E. coli* 622	-	50	Yolk of dead embryo, Qingdao, China (2017)
37	*E. coli* 623	low	80	Yolk of dead embryo, Yantai, China (2017)
38	*E. coli* 627	-	100	Yolk of dead embryo, Jinan, China (2017)
39	*E. coli* 721	low	80	Clinical dead broiler, Yantai, China (2018)
40	*E. coli* 722	Medium	80	Clinical dead broiler, Weifang, China (2018)
41	*E. coli* 723	-	50	1-day-old broiler, Qingdao, China (2017)
42	*E. coli* 724	-	50	Yolk of dead embryo, Weihai, China (2017)
43	*E. coli* 741	-	80	Ovary of culled broiler, Yantai, China (2020)
44	*E. coli* 744	-	60	Ovary of culled broiler, Qingdao, China (2020)
45	*E. coli* 864	-	50	Ovary of culled broiler, Qingdao, China (2021)
46	*E. coli* 871	-	60	Ovary of culled broiler, Weihai, China (2021)
47	*E. coli* 887	High	50	Ovary of culled broiler, Weifang, China (2018)
48	*E. coli* SD-C888	High	90	Ovary of culled broiler, Yantai, China (2018)
49	*E. coli* 889	Medium	80	Ovary of culled broiler, Yantai, China (2019)
50	*E. coli* 892	High	50	Ovary of culled broiler, Jinan, China (2019)
51	*E. coli* 895	High	50	Ovary of culled broiler, Weifang, China (2019)
52	*E. coli* 896	High	70	Ovary of culled broiler, Weihai, China (2019)
53	*E. coli* 897	low	70	Ovary of culled broiler, Weifang, China (2020)
54	*E. coli* 900	Medium	80	Ovary of culled broiler, Yantai, China (2021)
55	*E. coli* 905	-	50	Ovary of culled broiler, Weifang, China (2021)
56	*E. coli* 907	-	60	Ovary of culled broiler, Jinan, China (2021)
57	*E. coli* 7 l	Medium	70	Dead broiler in Jimo market, Qingdao, China (2021)
58	*E. coli* 9 l	High	80	Dead broiler in Jimo market, Qingdao, China (2022)

Efficiency of plating (EOP) values were determined by the ratio of the liberated phage particles of CE1 from each susceptible APEC strain to the PFUs from the indicator strain SD-C888. “High” stands for EOP ≥ 0.5, “Medium” stands for 0.1 ≤ EOP < 0.5, “Low” stands for 0.001 < EOP < 0.1, and “-” means EOP ≤ 0.001. Mortality rate of embryo is the lethality of each strain against 10 SPF broiler embryos, and it is considered a highly pathogenic strain when the mortality of chicken embryos exceeds 50% (Oh et al., 2012).

### 2.3. Phage isolation and purification

According to the previous report, phage isolation and purification were performed using double layer agar method (Zhang et al., 2013). A mixture of feces (5 g) and sterile saline (5 ml) was incubated at 37°C for 3 h, then centrifuged at 12000 r/min for 10 min. Mixture of the supernatant (1 ml) and host strain of SD-C888 (10^8^ CFU/ml,1 ml) in 100 ml LB broth were incubated overnight at 37°C with gentle shaking. After centrifugation at 12000 r/min for 10 min, the supernatant was filtered through a sterile disposable membrane filter (0.22 μm). Then, filtrate was mixed with SD-C888 at the same volume and incubated at 37°C for 5 min. Finally, the mixture (200 μl) was mixed well with molten agar (0.7%, 5 ml) before being overlaid on top of a 2% agar plate. Plates were incubated at 37°C for 12 h to form plaques. A well-isolated plaque was subjected to multiple rounds of plaque purification process.

### 2.4. Morphological observation

Morphology of phage CE1 was observed using a transmission electron microscope (HT7700, Hitachi, Japan) at an accelerating voltage of 80 kV as described previously (Ackermann, 2009).

### 2.5. Multiplicity of infection (MOI) and One-step growth curve

The optimal MOI was determined by mixing phage CE1 of different titers at various ratios (1, 0.1, 0.01, 0.001, 0.0001) with the host strain SD-C888 (10^8^ CFU/ml), and a bacterial culture without phage was used as control (Abedon, 2016). Each ratio was repeated in triplicate. A phage titer was determined by centrifugation at 12000 r/min for 10 min after incubation at 37°C for 3 h. Optimal MOI is determined by the ratio that produces the highest titer of phage.

One-step growth curve was performed as previously described with some modifications (Lee et al., 2021). Briefly, phage CE1 was mixed with the host strain SD-C888 at MOI 0.1, and incubated at 37°C for 5 min. To remove unabsorbed phages, the mixture was centrifuged at 12000 r/min for 30 s and washed twice with LB broth. The precipitate was resuspended in 5 ml LB broth, and incubated at 37°C with shaking at 180 r/min. Aliquots (200 μl) were taken at intervals of 5 min in the first hour, 20 min in the second hour, and 30 min in the third hour. Three aliquots were taken in triplicate each time. Phage titers were immediately detected by double-layer agar method. Burst size was calculated as the ratio of the final count of liberated phage particles to the initial count of phage particles (Sun et al., 2012).

### 2.6. Thermal and pH stability

For thermal stability, phage suspension (10^9^ PFU/ml) was incubated at 40°C, 50°C, 60°C, 70°C and 80°C for 1 h, and aliquots were taken at 20, 40 and 60 min for titer determination, respectively. For pH stability, we incubated CE1 suspension (10^9^ PFU/ml) in LB broth with various pH (2, 3, 4, 5, 6, 7, 8, 9, 10, 11, 12, 13) at 37°C for 1, 2 and 3 h. In triplicate, phage titers were determined by double-layer agar method.

### 2.7. Host range and efficiency of plating determination

Based on double-layer agar method, the host range of phage CE1 against 58 APEC strains was determined (Table 1). Efficiency of plating (EOP) values were determined by comparing the phage titer from tested APEC strain with that from SD-C888, a reference strain, and EOP from SD-C888 was considered as 1(Yin et al., 2022). According to the lytic capacity, EOP was divided into four categories: high production (EOP ≥ 0.5), medium production (0.1 ≤ EOP < 0.5), low production (0.001 < EOP < 0.1), and no production (EOP ≤ 0.001) (Khan and Nilsson, 2015).

### 2.8. Sequencing and genome analysis

The genomic DNA of CE1 was extracted according to the instructions of a Virus Genome DNA Extraction kit (CWBIO, Beijing, China). Then, the extracted DNA was verified with Nanodrop (Agilent 5,400, American), and the qualified DNA was sent to Genomics Solution Limited, SZHT (Shenzhen, China) for sequencing. The purified genomic DNA was sheared into c. 350 bp fragments to construct a paired-end (PE) library using the Nextera XT sample preparation kit (Illumina, San Diego, CA, United States). The PE reads of 150 bp were generated by a Novaseq 6,000 sequencer (Illumina, San Diego, CA, United States). High-quality reads were assembled into the phage genome using the *de novo* assembler SPAdes v.3.11.0 software (Bankevich et al., 2012). The complete sequence of CE1 was annotated using RAST (http://rast.nmpdr.org) and GeneMark (http://opal.biology.gatech.edu/GeneMark/) (Aziz et al., 2008; Besemer and Borodovsky, 2005). The predicted ORFs were verified using online BLASTP (http://www.ncbi.nlm.nih.gov/BLAST). Putative transfer RNA (tRNA) encoding genes were searched using tRNAscan-SE (http://trna.ucsc.edu/tRNAscan-SE/) (Schattner et al., 2005). A phylogenetic analysis was performed using MEGA 6.0 software based on the sequence of the entire genome, terminase large subunits, and the major capsid protein for CE1 (Tamura et al., 2013). The sequences used for comparation with phage CE1 were downloaded from NCBI, and a list of the accession numbers for the sequences were showed in Supplementary Table 1.

### 2.9. *In vitro* bactericidal activity

#### 2.9.1. Time-kill experiment under different MOIs

A series of experiments were conducted to determine the lytic efficiency of phage CE1 against *E. coli*. Firstly, the bactericidal activity of CE1 against host strain SD-C888 was assessed using optical densitometry and bacterial counting (Liu et al., 2022). Briefly, CE1 was cultured with SD-C888 (10^8^ CFU/ml) in LB broth at various MOIs (1, 0.1, 0.01 and 0.001), followed by incubation at 37°C with gentle shaking. A UV–vis spectrophotometer was used to measure the optical density at 600 nm in a 96-well plate at 1 h intervals for the first 10 h, and for 24 h. The bacterial growth was also monitored by measuring the bacteria titers at two-hour intervals for the first 10 h, and 24 h. A bacterial culture without phage was served as positive control, and LB broth as negative control. Each aliquot was tested three times.

#### 2.9.2. Spray disinfection effect

A spray disinfection test was conducted using CE1 suspension (10^8^ PFU/mL) in PBS which was continuously sprayed with humidifier in an airtight glass container. Afterwards, nutrient agar plates smeared with SD-C888 (10^8^ CFU/ml, 100 μl) were placed in the phage spray environment. A total of three plates were removed from the container at intervals of 10 min up to 90 min. No phage spraying plates were used as a control. Colonies on the plate are counted to determine the number of bacteria after all plates were incubated at 37°C for 24 h. Number of bacteria is calculated by taking the average of three parallel plates.

#### 2.9.3. Water disinfection effect

To determine the efficacy of CE1 in reducing bacteria in water samples, a final concentration of 10^6^ CFU/ml was achieved by adding SD-C888 suspensions to 0.9% sterile saline (100 ml) in two bottles. One bottle then received phage CE1 in a final concentration of 10^6^ PFU/mL, and the other bottle received 1 ml LB broth as control. Samples of 100 μl were taken every 2 h for 10 h from both bottles while they were kept at room temperature and shaken at 180 r/min. The experiment was performed in triplicate for each aliquot.

### 2.10. *In vivo* bactericidal activity

In order to test the bactericidal activity of CE1, a 50% lethal dose (LD_50_) of SD-C888 was administered to broilers. A total of fifty 1-day-old healthy broilers (Hubbards) were raised in isolators for 6 days. At the seventh day, they were randomly divided into five groups. Broilers in each group were treated with different dose of SD-C888 (10^9^, 10^8^, 10^7^, 10^6^, 10^5^ CFU, 0.1 ml) intramuscularly, then all groups were fed under the same condition and monitored at intervals of 2 h daily until 7 days after challenge. A total of one hundred 1-day-old healthy broilers (Hubbards) were raised in isolators for 6 days. At the seventh day, they were randomly divided into five groups (A ~ E) to test the protective effects of phage CE1. Groups A ~ C were challenged intramuscularly with SD-C888 (1.5 × 10^7^ CFU, 0.1 ml). An intramuscular inoculation of phage CE1 (10^8^ PFU, 0.1 ml) was administered to Group A at 2 h after challenge (Yang et al., 2015), whereas antibiotics (1.4 mg Linco-spectinomycin solution, 0.1 ml) were administered to Group B at 2 h after challenge. Group C received an intramuscular injection of PBS (0.1 ml) 2 h after bacterial inoculation as a challenged control. An intramuscular dose of CE1 (10^8^ PFU, 0.1 ml) was administered to group D without SD-C888 challenge, while LB (0.1 ml) was injected to group E as a blank control.

The health status of broilers was evaluated by scores at least three times per day, as previously described with some modifications (Chen et al., 2019). The health scores were divided into five categories (0: death; 1: near death; 2: difficult walking; 3: little diet and ruffled feathers; 4: decreased physical activity; 5: health). Protection rate was determined by counting the number of broilers who died or showed symptoms of disease in different challenged groups compared with the total number of broilers in each challenged group. Each group was randomly assigned three broilers for jugular blood collection at 24 h and 48 h after challenge. Subsequently, inflammatory cytokines of TNF-α, IL-1β, IFN-α, and IL-8 in the serums were detected using chicken cytokine ELISA kits (Nanjing Jiancheng Bioengineering Institute, Nanjing, China) in accordance with the instructions.

### 2.11. Statistical analysis

All assay results were visualized using GraphPad Prism 6.0. The general linear model procedure of SPSS, version 18.0 (SPSS Inc., Chicago, IL) was used to evaluate the main effects of phage treatment, antibiotic treatment, *E. coli* challenge, and their associated interaction. To determine whether there were significant differences between the groups, a one-way ANOVA was performed along with Duncan’s multiple comparison. Significance was set at *p* < 0.05, and a trend towards extremely significance at *p* < 0.01.

Nucleotide sequence accession number.

Complete genome sequence data of CE1 was deposited in the GenBank database, under accession number ON229909.

## 3. Results

### 3.1. Phage morphology

**Figure 1:**
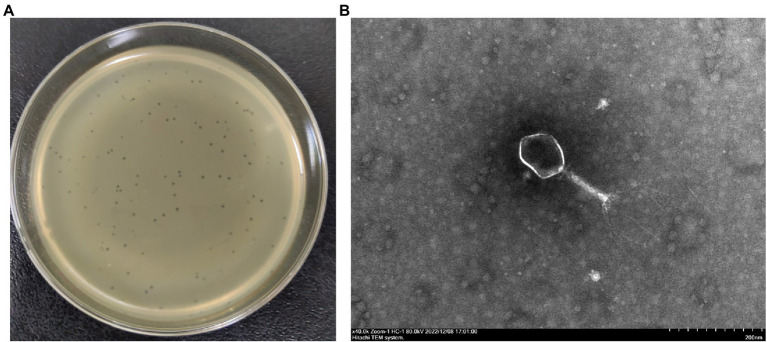
Morphological characteristics of phage CE1. Clear plaques produced by phage CE1 on an agar plate **(A)**; TEM image of phage CE1 consisting of an icosahedral head (80 ~ 100 nm in dimeter) and a retractable tail (120 nm in length) **(B)**.

The lytic phage vB_EcoM_CE1 (CE1) was isolated from broiler feces using *E. coli* SD-C888 as a host strain. It formed a clear round plaque about 1 mm in diameter after incubation at 37°C for 12 h on the double-layer agar plate (Figure 1A). TEM images showed that phage CE1 had an icosahedron head (80 ~ 100 nm in diameter) and a contracted tail (about 120 nm in length) (Figure 1B).

### 3.2. Biological characteristics of phage CE1

**Figure 2:**
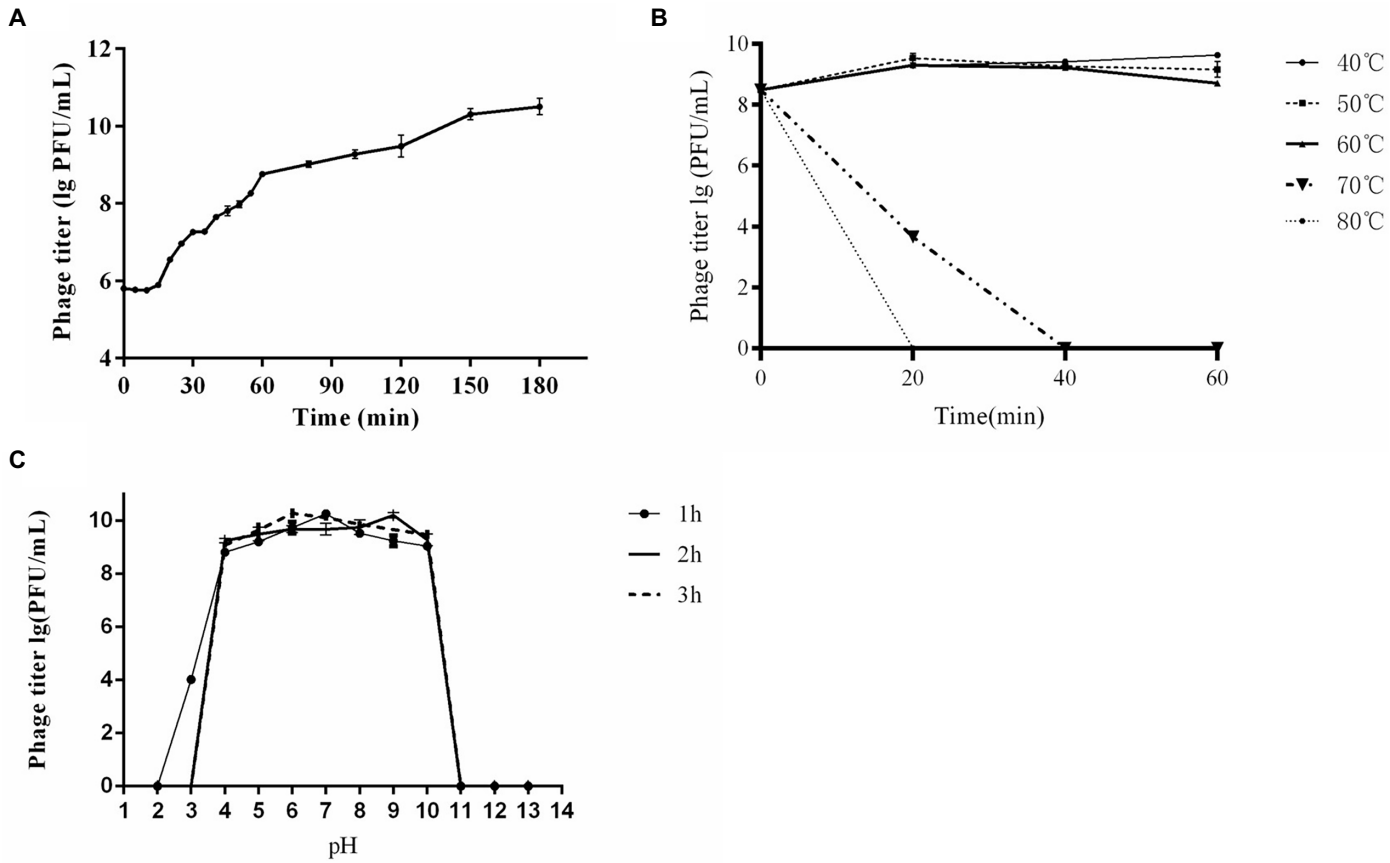
Biological properties of phage CE1. One-step growth curve of phage CE1. The data were expressed as means ± SD (n = 3) **(A)**; Sensitivity of CE1 to temperature. There was little effect on the activity of CE1 when the temperature varied from 40°C to 60°C for 1 h, while CE1 was quickly inactivated when the temperature rose to 70°C for 40 min or 80°C for 20 min. **(B)**; Sensitivity of CE1 to pH. Phage CE1 was relatively stable within the pH range of 4 to 10 when incubated at different pH values (2 to 13) for 3 h **(C)**.

At the MOIs of 0.01and 0.1, phage titer reached the highest value of ~10^10^ PFU/mL, indicating that the optimal MOI was 0.01 ~ 0.1. One-step growth curve showed that CE1 had a latent period of about 15 min, followed by rapid release of virus particles, and the final titer reached 3.2 × 10^10^ PFU/mL after a burst period of 135 min with a burst size of 63 PFUs/cell (Figure 2A). For thermal stability, the titers of phage CE1 showed no significant changes after incubation at 40°C, 50°C and 60°C for 60 min. However, phages were completely inactivated after 40 min of incubation at 70°C and 20 min of incubation at 80°C (Figure 2B), For pH stability, phage CE1 was stable over a pH range of 4 to 10 within 3 h (Figure 2C). The results indicated that phage CE1 was stable below 60°C for 1 h over the pH range of 4 to 10.

### 3.3. Host range and EOP of phage CE1

Based on the criteria of EOP values, phage CE1 could lyse 56.9% (33/58) of high pathogenic APEC strains listed in Table 1, of which phage CE1 had a high lytic activity of 20.7% (12/58), a medium lytic activity of 19.0% (11/58), and a low lytic activity of 17.2% (10/58), indicating that phage CE1 could potentially control colibacillosis caused by high pathogenic APEC strains due to its relatively wide host range.

### 3.4. Genomic features of phage CE1

The whole-genome sequence analysis indicated that phage CE1 had a linear double-stranded DNA, with a genome size of 167,955 bp and a GC content of 35.4%. A total of 271 ORFs accounting for 94.3% of the genome were identified, of which 225 were in the plus strand, while the rest were in the minus strand. There were 240 ORFs annotated as functional genes, including 172 structural genes, 59 transcription- and replication-related genes, 4 lysis-related genes, and 5 additive genes (Supplementary Table 2). The genome did not contain any genes associated with lysogenization, pathogenicity, or drug resistance. A total of eight tRNA genes have been identified, including tRNA^Gln^ (TTG), tRNA^Leu^ (TAA), tRNA^Gly^ (TCC), tRNA^Pro^ (TGG), tRNA^Ser^ (TGA), tRNA^Thr^ (TGT), tRNA^Met^ (CAT), and tRNA^Arg^ (TCT). The modular genomic structure of phage CE1 was similar to phage T4 (Miller et al., 2003) (Supplementary Figure 1).

### 3.5. Phylogenetic analysis of phage CE1

Comparative analysis of the whole-genome sequence indicated that CE1 had the highest DNA sequence identity (96.5% ~ 99.4%) to members of genus *Tequatrovirus*, including T4 and T4-like phages (Supplementary Figure 2). But there was some difference in genome characteristics among the phages (Supplementary Table 3). Phylogenetic trees based on the major capsid protein (Supplementary Figure 3) and terminase large subunit (Supplementary Figure 4) also revealed that all comparable phages belonged to the *Tequatrovirus* genus. Homologous phages were all of Enterobacteria phages, most of which were *E. coli* phages except for some *Yersinia* and *Shigella* phage. Based on the sequence, phylogenetic relationship, genomic size and architecture, phage CE1 was a new member of *Tequatrovirus* genus, *Straboviridae* family.

### 3.6. *In vitro* bactericidal activity of phage CE1

#### 3.6.1. *In vitro* bactericidal activity under different MOIs

**Figure 3:**
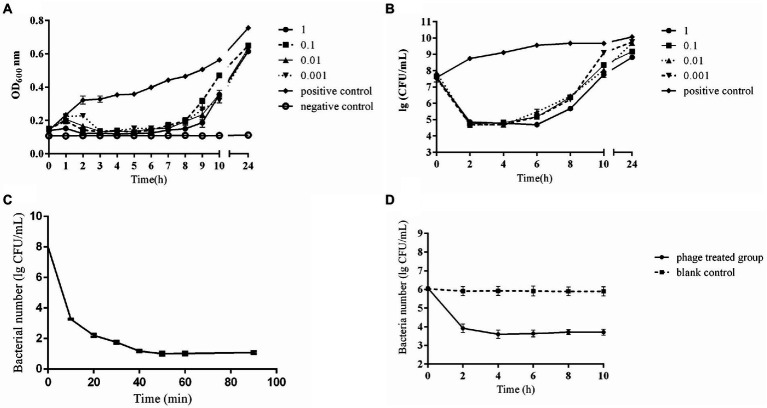
*In vitro* bactericidal activity of phage CE1. Detection of OD_600_ from phage CE1 against APEC strain SD-C888 under different MOIs (1, 0.1, 0.01, 0.001) **(A)**; Detection of CFUs from phage CE1 against APEC strain SD-C888 under different MOIs (1, 0.1, 0.01, 0.001) **(B)**; Detection of bactericidal activity in the spray disinfection **(C)**; Detection of bactericidal activity in water disinfection **(D)**. All data were expressed as means ± SD (n = 3).

*In vitro* bactericidal activities of phage CE1 against SD-C888 under various MOIs were shown in Figure 3. Within 24 h, the OD_600_ values of positive control increased continuously from 0.14 to 0.75, while that of negative control remained unchanged. Growth of SD-C888 was completely inhibited at all MOIs after treatment with CE1 between 2 ~ 6 h. However, OD_600_ values gradually rose from 6 h after phage treatment. After 24 h, there was a significant difference in the OD_600_ values(*p* < 0.05)compared with the positive control, and no significant difference in MOI values (*p* > 0.05) (Figure 3A). A rapid decline in SD-C888 numbers was observed during the first 2 h (from 5 × 10^7^ CFU/ml to no more than 10^5^ CFU/ml), followed by an equilibrium during 2 ~ 4 h of different MOIs. After that, it began to rise gradually until it reached 24 h (Figure 3B). Although the bacterial number of all MOIs was over 10^8^ CFU/ml at 24 h, there was a significant difference of MOI 1 and 0.1 compared with positive control (*p* < 0.05). This demonstrated that CE1 could significantly inhibit bacterial growth under suitable MOI, and the highest bactericidal activity was observed at MOI 1.

#### 3.6.2. Spray disinfection effect

As a result of the spray disinfection test, phage CE1 showed a gradual decrease in bacterial number against SD-C888. There was a rapid reduction in concentration of SD-C888 (from 1.2 × 10^6^ CFU/ml to 5.2 × 10^3^ CFU/ml) within 40 min, then the number maintained a balance until 90 min (Figure 3C), which suggested that CE1 could be used for spray disinfection.

#### 3.6.3. Water disinfection effect

As shown in Figure 3D, phage CE1 treatment reduced bacterial load by more than 2 log in the first 2 h of incubation, and was maintained for 2–10 h. There was a significant difference compared with the blank control (*p* < 0.01), which indicated the potential of CE1 as a biocontrol agent against APEC strains present in water.

### 3.7. Protection of phage CE1 in a broiler challenged model

A broiler challenged model against *E. coli* SD-C888 was established at LD_50_ of 1.5 × 10^7^ CFU calculated by Reed and Muench method.

After the experiment, health scores were calculated and compared among groups. Scores of the challenged group were significantly lower than those of the other groups (*p* < 0.0001), while no significant difference was seen in scores of the other groups (*p* > 0.05) (Supplementary Figure 5). The challenged broilers showed severe clinical signs of difficulty in walking or swollen joints, of which eleven died within seven days. While no death was observed in challenged broilers treated with phage CE1, only one broiler showed signs of loose feathers. The antibiotic-treated group with Linco-spectinomycin had three broilers with signs of illness and one death (Table 2). In addition, CE1 (10^8^ PFU) did not have any side effects on broilers. These results indicated that phage CE1 was effective in treating chicken colibacillosis.

**Table 2:** Protection of phage CE1 on challenged broilers.

Groups	Dose (0.1 ml)		Broiler number		
	Phage or antibiotics	E. coli SD-C888	Symptoms ^a^	Death	protection rate
A (phage treated)	10^8^ PFU	1.5 × 10^7^ CFU	1	0	19/20
B (Linco-spectinomycin treated)	1.4 mg	1.5 × 10^7^ CFU	3	1	16/20
C (challenge control)	10^8^ PFU	1.5 × 10^7^ CFU	5	11	4/20
D (phage control)	10^8^ PFU		0	0	20/20
E (blank control)	0.1 ml LB		0	0	20/20

a Broiler had symptoms of decreased activity and diet, loose feather, difficulty in walking, or swollen joints.

**Figure 4:**
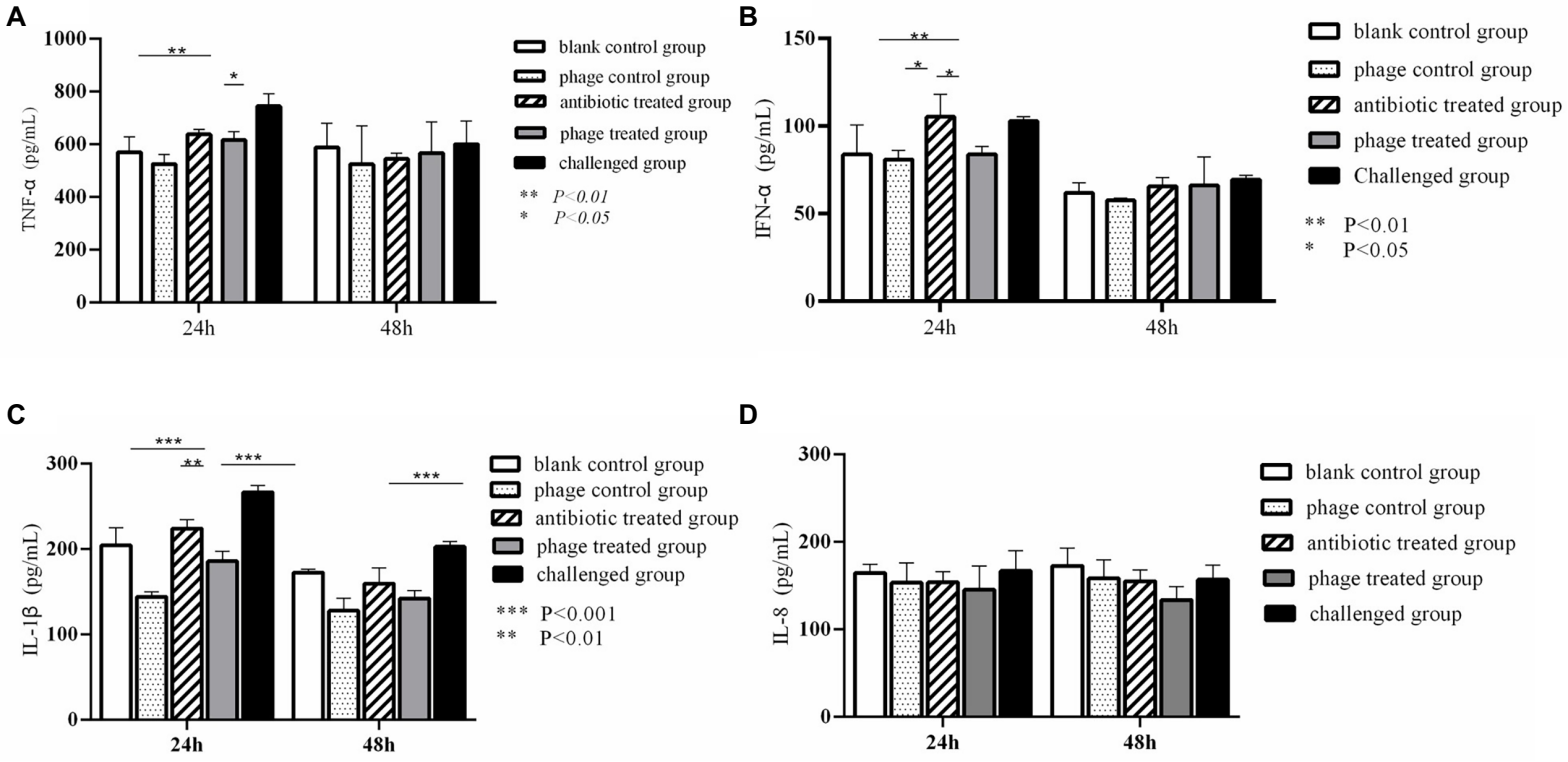
Dynamic change of cytokines in the animal experiment. Levels of TNF-α in different group. **P < 0.01, *P < 0.05 **(A)**; Levels of IFN-α in different group. **p < 0.01, *p < 0.05 **(B)**; Levels of IL-1β in different group. **p < 0.01, ***P < 0.001 **(C)**; Levels of IL-8 in different group **(D)**.

As shown in Figure 4A, TNF-α levels in the phage treated group and antibiotics treated group were significantly lower than those in the challenged group at 24 h (*P*<0.05). the challenged group reached a significant increase in TNF-α compared to the blank control group at 24 h (*P<*0.01). No significant differences of TNF-α were observed between any of the other groups at 24 h and 48 h (*P*>0.05). It was found that the challenged group significantly increased IFN-α compared with the blank control group (*P<*0.01) and phage treated group (*P*<0.05) at 24 h. There was no difference between the phage treated group and the blank control group at 24 h (*p* > 0.05), and no difference between all groups at 48 h (*p* > 0.05) (Figure 4B). Figure 4C showed that challenged group significantly increased IL-1β comparing with phage treated group, antibiotic- treated group, and blank control group at 24 h (*p* < 0.01). In addition, there was no difference between phage treated group and blank control group at 24 h (*p* > 0.05). Moreover, there was a significant difference between the phage treated group and the challenged group at 48 h (*p* < 0.001), and no difference was seen in the other groups (*p* > 0.05). Furthermore, no difference was found in IL-8 between all groups at 24 h and 48 h (*p* > 0.05) (Figure 4D).

## 4. Discussion

There has been a great deal of interest in phage therapy against colibacillosis caused by APEC due to the increasing number of multi-drug resistant strains of *E. coli*. In the current study, a lytic phage CE1 was isolated from broiler feces, and exhibited a broad host range (56.9%) and high titer (10^10^ PFU/mL). Additionally, it remained stable at wide pHs (4 to 10) for 3 h and temperatures (40°C to 60°C) for 60 min, indicating that it could survive in a wide range of environmental conditions. It is possible to obtain the maximum benefit by adjusting phage amounts in future industrial processes by measuring the MOI and one-step growth curve (O’Flynn et al., 2004). The extremely low optimal MOI (0.1 to 0.01), short incubation period (15 min), and large burst size (63 PFUs/cell) indicated that phage CE1 has high proliferation efficiency and lytic activity. Thus, it could be used as a backup strain for phage therapy.

It is known that the host range of phage is closely associated with the interaction between receptor recognition proteins on their tails and bacterial receptor proteins. Host specificity of CE1 might be attributed to its distal tail fiber gp37 (ORF12) and gp38 (ORF13) protein, and the N-terminal region of gp37 that interacts with gp36 (ORF11) is structurally conserved between CE1 and those of the known T-even phages (Yu et al., 2000). It was found that the amino acid identities of gp36 were 100% homologous between CE1 and T4-like phages of HY01. According to early studies, CE1 could have similar characteristics with phage HY01 (Lee et al., 2016), and our test confirmed this. Additionally, CE1 lysis-associated RI gene (ORF150) had been reported to act as a cleavable signal peptide (Tran et al., 2007), while gene RIIA (ORF255) possessed 97.93% homology to T4, they both contributed to the lyse of CE1 against host bacteria. As tRNA genes corresponded to codons used by phages rather than hosts, they improved phage replication and host translation efficiency. (Bailly-Bechet et al., 2007; Rak et al., 2018), this was also confirmed by previous studies of T4-like phage (Liao et al., 2011). Since a major disadvantage of phage therapy is the spread of virulence genes and drug resistance genes among bacteria, the safety of CE1 was confirmed by whole-genome sequence. Antimicrobial activity of CE1 against exponentially growing planktonic cells was evaluated using a time-kill assay. CE1 significantly limited bacteria growth within 4 h of incubation at MOI of 0.001 to 1, with resistant bacteria gradually increasing. Phage therapy faces the threat of phage-resistant variants. Although this may pose a barrier to application, early studies have shown that resistant variants tend to be less virulent, and are easily cleared by phagocytes and innate immune systems (Gu et al., 2012). This can also reduce drug resistance and increase the efficiency of phages by cocktail phages (Clavijo et al., 2019; Poojari et al., 2022). In view of the fact that APEC could flood all over a breeding environment, mainly invading the respiratory tract and alimentary tract, phage spray disinfection is a good option for biocontrol. The application of bacteriophage-containing aerosol against *Mycobacterium tuberculosis* had been reported to be effective (Tseng et al., 2019). In our study, statistical differences were found in bacterial counts, with great decrease (10^6^ CFU) after CE1 sprayed for 40 min. Additionally, CE1 was also effective on reducing bacterial numbers in water environments. *Salmonella enteritidis* reduced in environmental samples and fecal samples in layer farms by using autophages (bacteriophages isolated from the same environment as the pathogen) (Sevilla-Navarro et al., 2018). CE1 was not limited to be used as an autophage because of the wide host range. The characteristics of CE1 suggested its possibility as an effective and non-pungent disinfectant used in broiler farms to control colibacillosis.

Recent studies have demonstrated that phage therapy is effective against respiratory infections, long-term, persistent, or chronic bacterial infections (Abedon, 2019; Chang et al., 2018). Our experiment on broilers showed that phage CE1 (10^8^ PFU) reduced bacterial populations to levels that may allow the host immune response to mount a successful defense and clear the infection. Intramuscular administration is the fastest way to create a pathogenic model. In our animal experiment, we successfully obtained the pathogenic model through intramuscular injection. During broiler breeding, antibiotic is logically not possible to use through intramuscular administration because of the intensive breeding of broilers. While antibiotics can be used with inactivated vaccines through intramuscular injection, bacteriophage can be used as substitutes for antibiotics together with the inactivated vaccines, and our previous test has shown that the effect of injection is better than that of oral administration. Based on the results of the current experiment, a single dose of CE1 (10^8^ PFU) intramuscular administration provided superior protection over Linco-spectinomycin (1.4 mg). As compared to the control, inflammatory cytokines of TNF-α, IFN-α, IL-1β increased dramatically after bacterial challenge at 24 h. After administration of phage CE1, cytokine levels decreased to almost normal levels.

Despite the fact that phage CE1 could be able to control APEC-associated infections in the present study, there are still some limitations of the therapeutic effect of phage CE1, including different routes, doses, intervals, phage cocktails, and phage dynamics *in vivo*. Further studies will be conducted in the future to address these details.

## 5. Conclusion

Our study described the main biological characteristics of an isolated *E. coli* phage CE1 with a wide host range. The genetic evolution analysis of CE1 revealed that it was a member of the *Tequatrovirus* genus, *Straboviridae* family. A functional annotation of the whole-genome revealed no genes related to resistance, virulence, or lysogeny. *In vitro*, CE1 was effective on inhibiting *E. coli*. Compared to antibiotics, CE1 provided better protection against broiler challenge. Our results highlight the potential of phage CE1 in the treatment of colibacillosis and as an environmentally friendly disinfectant. These results suggest phage CE1 might be a promising strain for combating *E. coli* infections both *in vitro* and *in vivo*. Nevertheless, further tests are needed to confirm its effects in the future.

## Data availability statement

The datasets presented in this study can be found in online repositories. The names of the repository/repositories and accession number(s) can be found in the article/Supplementary material.

## Ethics statement

The animal study was reviewed and approved by Research Ethics Committee at Qingdao Agricultural University, Shandong, China.

## Author contributions

WL designed the study and prepared the manuscript. ZT executed characteristic of phage. NT contributed to the bactericidal activity of phage *in vitro*. XW engaged in animal experiments. HR helped with the manuscript. CZ analyzed the whole-genome sequence. LZ was in charge of reagents and instruments supply. LH conducted some data analysis. LG participated in the experiment design. All authors were responsible for data integrity and accuracy of the analysis, contributed to the article, and approved the submitted version.

## Funding

This work was supported by a grant from Youth Innovation Team Project for Talent Introduction and Cultivation in Universities of Shandong Province (2021).

## Conflict of interest

LG was employed by Shandong Yisheng Livestock & Poultry Breeding Co., Ltd.

The remaining authors declare that the research was conducted in the absence of any commercial or financial relationships that could be construed as a potential conflict of interest.

## Publisher’s note

All claims expressed in this article are solely those of the authors and do not necessarily represent those of their affiliated organizations, or those of the publisher, the editors and the reviewers. Any product that may be evaluated in this article, or claim that may be made by its manufacturer, is not guaranteed or endorsed by the publisher.
