# Supplementary material for: Characterization of a lytic Escherichia coli phage CE1 and its potential use in therapy against avian pathogenic Escherichia coli infections
Source: Front Microbiol. 2023 Feb 16;14:1091442. doi: 10.3389/fmicb.2023.1091442 (PMC9978775; doi:10.3389/fmicb.2023.1091442)
Supplement: Supplementary file 1 [file Table_1.DOCX]

| Phage | Accession number |
| --- | --- |
| Escherichia phage T4_ev240 | LR597657.1 |
| Escherichia phage JLBYU31 | OK272473.1 |
| Escherichia phage T4 | MT984581.1 |
| Enterobacteria phage RB6 | KM606996.1 |
| Escherichia phage AR1 | AP011113.1 |
| Escherichia phage vb_EcoM_bov10K1 | MT884007.2 |
| Escherichia phage 132 | MZ417521.1 |
| Yersinia phage fPS-2 | NC_054943.1 |
| Enterobacteria phage T4 strain wild | KJ477684.1 |
| Escherichia phage vB_EcoM_Nami | MZ502380.1 |
| Citrobacter phage PhiZZ23 | NC_054901.1 |
| Escherichia phage vB_EcoM_IME537 | NC_054921.1 |
| Escherichia phage vB_EcoM-101112UKE3-1 | MZ234013.1 |
| Enterobacteria phage SV76 | OM339528.1 |
| Escherichia phage vB_EcoM-fHoEco02 | MG781191.1 |
| Escherichia phage APTC-EC-2A | OK274152.1 |
| Escherichia phage vB_EcoM_SYGD1 | MW883059.1 |
| Escherichia phage vB_EcoM-S1P5QW | OL956808.1 |
| Shigella phage pSs-1 | NC_025829.1 |
| Shigella phage CM8 | NC_054939.1 |
| Escherichia phage HY03 | KR269718.1 |
| Escherichia phage vB_vPM_PD112 | NC_054928.1 |
| Salmonella phage pSe_SNUABM_01 | NC_054937.1 |
| Shigella phage Sf21 | YP_009618983.1 |
| Escherichia phage vB_EcoM_FT | QLF81042.1 |
| Escherichia phage KIT03 | YP_010071646.1 |
| Yersinia phage phiD1 | YP_009149417.1 |
| Shigella phage A2 | UVD36852.1 |
| Escherichia phage vB_EcoM_ACG-C40 | YP_006986718.1 |

Table S1 A list of the accession numbers for the sequences used in the phylogenetic analysis
