# Supplementary material for: Characterization of a lytic Escherichia coli phage CE1 and its potential use in therapy against avian pathogenic Escherichia coli infections
Source: Front Microbiol. 2023 Feb 16;14:1091442. doi: 10.3389/fmicb.2023.1091442 (PMC9978775; doi:10.3389/fmicb.2023.1091442)
Supplement: Supplementary file 2 [file Table_2.DOCX]

Table S2 Summary of phage CE1 ORFs with predicted functions

|  | start | stop | strand | function | Scientific Name | E value |  | Accession |
| --- | --- | --- | --- | --- | --- | --- | --- | --- |
| ORF1 | 8 | 337 | + | putative anti-restriction nuclease  [Escherichia phage JLBYU31] | Escherichia phage JLBYU31 | 1E-74 | 99.08% | UGO54985.1 |
| ORF2 | 334 | 795 | + | hypothetical protein | Escherichia phage ECML-134 | 3E-111 | 100.00% | YP_009102730.1 |
| ORF3 | 795 | 1091 | + | hypothetical protein | Enterobacteria phage T6 | 5E-66 | 100.00% | YP_010067405.1 |
| ORF4 | 1162 | 1293 | + | hypothetical protein | Escherichia phage vB_EcoM_WFL6982 | 2E-23 | 100.00% | QBQ77012.1 |
| ORF5 | 1377 | 1655 | + | Phage anti-restriction nuclease | Escherichia phage wV7 | 6E-46 | 100.00% | YP_007004994.1 |
| ORF6 | 1652 | 1804 | + | hypothetical protein | Escherichia phage vB_vPM_PD112 | 4E-26 | 100.00% | YP_010072922.1 |
| ORF7 | 1817 | 2089 | + | Phage anti-sigma factor | Escherichia virus T4 | 2E-57 | 100.00% | 6K4Y_I |
| ORF8 | 2746 | 2090 | - | Phage holin | Escherichia phage vB_vPM_PD112 | 1E-160 | 100.00% | YP_010072920.1 |
| ORF9 | 3572 | 2778 | - | tail fibers protein [Escherichia phage vB_EcoM_F1] | Escherichia phage vB_EcoM_F1 | 1E-104 | 97.35% | YP_010068739.1 |
| ORF10 | 6927 | 3604 | - | Phage tail fiber | Escherichia phage vB_vPM_PD112 | 0 | 90.88% | YP_010072918.1 |
| ORF11 | 7592 | 6936 | - | Phage tail fibers | Escherichia phage HY01 | 3E-156 | 100.00% | YP_009148688.1 |
| ORF12 | 8770 | 7655 | - | Phage long tail fiber | Escherichia phage vB_vPM_PD112 | 0 | 100.00% | YP_010072916.1 |
| ORF13 | 12648 | 8779 | - | Phage long tail fiber | Escherichia phage vB_vPM_PD112 | 0 | 100.00% | YP_010072915.1 |
| ORF14 | 12751 | 13668 | + | Phage ribonuclease H (EC 3.1.26.4) | Escherichia phage vB_EcoM_IME537 | 0 | 99.67% | YP_010071013.1 |
| ORF15 | 13677 | 13946 | + | putative double-stranded DNA-binding protein [Escherichia phage U115] | Escherichia phage U115 | 3E-55 | 98.88% | UAV89206.1 |
| ORF16 | 13924 | 14262 | + | late promoter transcription accessory protein [Escherichia phage vB_EcoM_IME537] | Escherichia phage vB_EcoM_IME537 | 9E-61 | 100% | YP_010071011.1 |
| ORF17 | 14259 | 14912 | + | loader of DNA helicase [AcinetobacterphageAM101] [Escherichia phage vB_Eco_NR1] | Escherichia phage vB_Eco_NR1 | 3E-154 | 99.54% | CAD7712067.1 |
| ORF18 | 15012 | 15920 | + | Single stranded DNA-binding protein,  phage-associated | Escherichia phage vB_vPM_PD112 | 0 | 100.00% | YP_010072910.1 |
| ORF19 | 16067 | 16294 | + | hypothetical protein CPTSV76_036  [Enterobacteria phage SV76] | Enterobacteria phage SV76 | 1E-46 | 98.67% | ULF50299.1 |
| ORF20 | 16340 | 16726 | + | Phage protein | Escherichia phage vB_vPM_PD112 | 5E-86 | 100.00% | YP_010072908.1 |
| ORF21 | 16781 | 17023 | + | hypothetical protein [Shigella phage Sfk20] | Shigella phage Sfk20 | 5E-51 | 98.75% | QPP47021.1 |
| ORF22 | 17034 | 17276 | + | Phage protein | Escherichia phage YUEEL01 | 8E-50 | 98.75% | YP_010074955.1 |
| ORF23 | 17280 | 17864 | + | Dihydrofolate reductase, phage-associated | Shigella phage pSs-1 | 7E-131 | 100.00% | YP_009111050.1 |
| ORF24 | 17861 | 18067 | + | hypothetical protein | Shigella phage pSs-1 | 4E-43 | 100.00% | YP_009111049.1 |
| ORF25 | 18067 | 18618 | + | Thymidylate synthase (EC 2.1.1.45) | Escherichia phage vB_vPM_PD112 | 9E-134 | 100.00% | YP_010072903.1 |
| ORF26 | 18875 | 19189 | + | Thymidylate synthase (EC 2.1.1.45) | Escherichia phage vB_vPM_PD112 | 5E-70 | 100.00% | YP_010072902.1 |
| ORF27 | 19213 | 19476 | + | hypothetical protein LDJ77_00070 [Escherichia phage UoN_LDJ77_1] | Escherichia phage UoN_LDJ77_1 | 6E-56 | 98.85% | UFK27318.1 |
| ORF28 | 19747 | 22011 | + | Ribonucleotide reductase of class Ia (aerobic),  alpha subunit (EC 1.17.4.1) | Escherichia phage vB_vPM_PD112 | 0 | 100.00% | YP_010072900.1 |
| ORF29 | 22063 | 22740 | + | Ribonucleotide reductase of class Ia (aerobic),  beta subunit (EC 1.17.4.1) | Escherichia phage vB_vPM_PD112 | 8E-167 | 100.00% | YP_010072899.1 |
| ORF30 | 23066 | 23545 | + | Ribonucleotide reductase of class Ia (aerobic),  beta subunit (EC 1.17.4.1) | Escherichia phage vB_EcoM_112 | 6E-116 | 100.00% | YP_009030835.1 |
| ORF31 | 23573 | 23983 | + | aerobic NDP reductase small subunit  [Escherichia phage vB_EcoM_112] | Escherichia phage vB_EcoM_112 | 7E-116 | 100.00% | YP_009030835.1 |
| ORF32 | 24036 | 25160 | + | RNA ligase | Escherichia phage vB_EcoM_DalCa | 0 | 100.00% | YP_010067642.1 |
| ORF33 | 25222 | 25728 | + | Putative phage alc transcription terminator  (ACLAME 1242) | Escherichia phage vB_EcoM_IME537 | 7E-122 | 100.00% | YP_010070993.1 |
| ORF34 | 25719 | 26072 | + | hypothetical protein | Escherichia phage vB_vPM_PD112 | 1E-45 | 100.00% | YP_010072894.1 |
| ORF35 | 26069 | 26368 | + | hypothetical protein KMC11_gp188  [Escherichia phage vB_EcoM_G4507] | Escherichia phage vB_EcoM_G4507 | 2E-66 | 98.99% | YP_010070602.1 |
| ORF36 | 26365 | 26595 | + | Phage protein | Shigella phage Sf22 | 6E-49 | 100.00% | YP_009614872.1 |
| ORF37 | 26592 | 26894 | + | Phage protein | Escherichia phage vB_vPM_PD112 | 6E-66 | 100.00% | YP_010072891.1 |
| ORF38 | 26891 | 27799 | + | AAA family ATPase  [Escherichia phage vB_vPM_PD112] | Escherichia phage vB_vPM_PD112 | 0 | 99.34% | YP_010072890.1 |
| ORF39 | 27799 | 27996 | + | Phage protein | Escherichia phage HY01 | 3E-40 | 100.00% | YP_009148659.1 |
| ORF40 | 27989 | 28189 | + | Phage protein | Escherichia phage vB_EcoM-UFV13 | 1E-38 | 100.00% | YP_009290482.1 |
| ORF41 | 28192 | 28467 | + | hypothetical protein KMB98_gp194 [Enterobacteria phage RB18] | Enterobacteria phage RB18 | 3E-58 | 98.90% | YP_010067098.1 |
| ORF42 | 28530 | 28766 | + | hypothetical protein [Escherichia phage EP01] | Escherichia phage EP01 | 7E-48 | 98.72% | UIU46963.1 |
| ORF43 | 28763 | 29101 | + | Phage protein | Escherichia phage vB_EcoM_G2540-3 | 2E-74 | 98.21% | QBO65624.1 |
| ORF44 | 29098 | 29679 | + | deoxycytidylate deaminase  [Escherichia phage MLP2] | Escherichia phage MLP2 | 1E-137 | 97.93% | EEX9355338.1 |
| ORF45 | 29679 | 29915 | + | gp31.2 hypothetical protein [Escherichia phage PP01] | Escherichia phage PP01 | 5E-49 | 98.72% | YP_010073587.1 |
| ORF46 | 29916 | 30224 | + | SH3 beta-barrel fold-containing protein [Enterobacteria phage RB51] | Enterobacteria phage RB51 | 2E-66 | 99.02% | YP_002854166.1 |
| ORF47 | 30281 | 30616 | + | co-chaperone GroES family protein [Serratia phage PhiZZ30] | Serratia phage PhiZZ30 | 9E-73 | 99.10% | WP_015969375.1 |
| ORF48 | 30764 | 31012 | + | rIII lysis inhibitor [Escherichia phage teqdroes] | Escherichia phage teqdroes | 5E-51 | 98.78% | YP_010074086.1 |
| ORF49 | 31091 | 31309 | + | 1,4-alpha-glucan (glycogen) branching enzyme,  GH-13-type (EC 2.4.1.18) | Escherichia virus T4 | 3E-31 | 100.00% | P17310.1 |
| ORF50 | 31420 | 31752 | + | hypothetical protein KMC00_gp204  [Escherichia phage vB_EcoM_DalCa] | Escherichia phage vB_EcoM_DalCa | 5E-73 | 98.18% | YP_010067623.1 |
| ORF51 | 31821 | 32186 | + | Phage protein | Escherichia virus RB14 | 8E-86 | 100.00% | YP_002854540.1 |
| ORF52 | 32227 | 32514 | + | Phage protein | Shigella phage pSs-1 | 1E-63 | 98.95% | YP_009111022.1 |
| ORF53 | 32514 | 32711 | + | hypothetical protein KMC09_gp204 [Escherichia phage vB_EcoM_G50] | Escherichia phage vB_EcoM_G50 | 2E-37 | 98.46% | YP_010070031.1 |
| ORF54 | 32708 | 32914 | + | Phage protein | Escherichia phage vB_EcoM_G50 | 4E-42 | 100.00% | YP_010070030.1 |
| ORF55 | 32907 | 33365 | + | hypothetical protein | Shigella phage CT01 | 6E-108 | 99.34% | UDY80594.1 |
| ORF56 | 33362 | 34201 | + | Phage protein (ACLAME 784) | Escherichia phage vB_vPM_PD112 | 0 | 100.00% | YP_010072872.1 |
| ORF57 | 34201 | 34470 | + | Phage protein | Enterobacteria phage vB_EcoM_IME340 | 4E-60 | 98.88% | YP_010066455.1 |
| ORF58 | 34467 | 35927 | + | DNA ligase, phage-associated | Escherichia phage vB_vPM_PD112 | 0 | 100.00% | YP_010072870.1 |
| ORF59 | 35924 | 36112 | + | Phage protein | Yersinia phage phiD1 | 2E-36 | 100.00% | YP_009149441.1 |
| ORF60 | 36165 | 38213 | + | Putative RNA polymerase-ADP- ribosyltransferase Alt (ACLAME 423) | Escherichia phage vB_vPM_PD112 | 0 | 100.00% | YP_010072868.1 |
| ORF61 | 38399 | 38518 | + | hypothetical protein | Escherichia phage vB_EcoM_IME537 | 3E-16 | 100.00% | YP_010070964.1 |
| ORF62 | 38634 | 38924 | + | hypothetical protein KMC25_gp213  [Escherichia phage teqhad] | Escherichia phage teqhad | 6E-60 | 98.96% | YP_010074397.1 |
| ORF63 | 39918 | 38953 | - | tail assembly protein | Escherichia phage RB3 | 0 | 99.38% | YP_009098578.1 |
| ORF64 | 41012 | 39918 | - | Phage baseplate tail tube cap  (T4-like gp48) | Shigella phage SHBML-50-1 | 0 | 99.45% | YP_009288557.1 |
| ORF65 | 42793 | 41021 | - | Phage baseplate hub | Escherichia phage vB_EcoM_KAW1E185 | 0 | 100.00% | YP_010071396.1 |
| ORF66 | 43248 | 42790 | - | baseplate hub distal subunit  [Escherichia phage KarlGJung] | Escherichia phage KarlGJung | 3E-92 | 100.00% | QXV82008.1 |
| ORF67 | 44443 | 43268 | - | base plate hub subunit [Tequatrovirus RB14] | Tequatrovirus RB14 | 0 | 99.74% | YP_002854524.1 |
| ORF68 | 45192 | 44443 | - | Phage baseplate | Escherichia phage slur07 | 0 | 100.00% | YP_009197267.1 |
| ORF69 | 45243 | 45869 | + | Phage baseplate hub | Escherichia phage vB_vPM_PD112 | 6E-150 | 100.00% | YP_010072859.1 |
| ORF70 | 45869 | 46267 | + | baseplate wedge subunit [Shigella phage ESh36] | Shigella phage ESh36 | 1E-89 | 99.24% | URY16176.1 |
| ORF71 | 46334 | 46747 | + | Single stranded DNA-binding protein,  phage-associated | Escherichia phage slur14 | 7E-94 | 100.00% | YP_009180685.1 |
| ORF72 | 46747 | 46971 | + | Phage protein | Escherichia phage vB_EcoM_OE5505 | 1E-46 | 100.00% | YP_010072474.1 |
| ORF73 | 47000 | 47167 | + | DUF2685 domain-containing protein  [Escherichia phage T4] | Escherichia phage T4 | 8E-31 | 100.00% | UJD20394.1 |
| ORF74 | 47453 | 47223 | - | Phage DNA helicase | Escherichia phage vB_EcoM_IME537 | 5E-36 | 100.00% | YP_010070951.1 |
| ORF75 | 48990 | 47479 | - | Phage DNA helicase | Escherichia phage vB_vPM_PD112 | 0 | 99.80% | YP_010072853.1 |
| ORF76 | 49041 | 49721 | + | Inh inhibitor of gp21 prohead protease | Escherichia phage UFV-AREG1 | 2E-151 | 100.00% | YP_009281514.1 |
| ORF77 | 49731 | 50222 | + | capsid and scaffold protein | Escherichia phage vB_vPM_PD112 | 6E-94 | 98.16% | YP_010072851.1 |
| ORF78 | 50300 | 50860 | + | capsid and scaffold protein | Escherichia phage vB_vPM_PD112 | 3E-133 | 100.00% | YP_010072851.1 |
| ORF79 | 50961 | 51164 | + | Phage protein | Escherichia phage vB_EcoM_112 | 3E-39 | 100.00% | YP_009030785.1 |
| ORF80 | 51151 | 51429 | + | hypothetical protein G2540_00181 [Escherichia phage vB_EcoM_G2540] | Escherichia phage vB_EcoM_G2540 | 2E-59 | 98.91% | QBO63415.1 |
| ORF81 | 51439 | 52443 | + | RNA ligase | Escherichia phage vB_EcoM_IME537 | 0 | 100.00% | YP_010070944.1 |
| ORF82 | 53756 | 52473 | - | capsid vertex protein [Escherichia phage EC128] | Escherichia phage EC128 | 0 | 99.53% | URO83694.1 |
| ORF83 | 55405 | 53840 | - | major capsid protein | Shigella phage CT01 | 0 | 99.81% | UDY80567.1 |
| ORF84 | 56233 | 55424 | - | Phage prohead assembly (scaffolding)  protein | Shigella phage Shfl2 | 1E-134 | 100.00% | YP_004415066.1 |
| ORF85 | 56902 | 56264 | - | prohead core scaffolding protein and protease [Escherichia phage T4] | Escherichia phage T4 | 8E-152 | 99.53% | NP_049785.1 |
| ORF86 | 57327 | 56902 | - | prohead core protein [Escherichia phage slur14] | Escherichia phage slur14 | 2E-78 | 100.00% | YP_009180671.1 |
| ORF87 | 57569 | 57327 | - | Phage prohead core protein | Escherichia phage vB_EcoM-101112UKE3-1 | 2E-21 | 100.00% | QZI78910.1 |
| ORF88 | 59143 | 57569 | - | Phage portal vertex of the head | Escherichia phage vB_EcoM_IME537 | 0 | 99.81% | YP_010070937.1 |
| ORF89 | 59718 | 59227 | - | Phage BASE fibers | Escherichia phage ECML-134 | 4E-117 | 100.00% | YP_009102639.1 |
| ORF90 | 60431 | 59826 | - | gp19.1 1 | Escherichia phage ECML-134 | 3E-145 | 100.00% | YP_009102638.1 |
| ORF91 | 62454 | 60475 | - | Phage tail sheath | Escherichia phage vB_vPM_PD112 | 0 | 99.54% | YP_010072838.1 |
| ORF92 | 63871 | 62486 | - | Phage terminase, large subunit | Escherichia phage HY03 | 0 | 100.00% | YP_009284027.1 |
| ORF93 | 64458 | 63859 | - | hypothetical protein | Escherichia phage vB_vPM_PD112 | 5E-146 | 100.00% | YP_010072836.1 |
| ORF94 | 65338 | 64445 | - | Phage terminase, large subunit | [Escherichia phage EC121](https://www.ncbi.nlm.nih.gov/Taxonomy/Browser/wwwtax.cgi?id=2025815) | 0 | 100.00% | [YP_010067846.1](https://www.ncbi.nlm.nih.gov/protein/YP_010067846.1?report=genbank&log$=prottop&blast_rank=4&RID=DPPWJ2KN013) |
| ORF95 | 65816 | 65322 | - | small terminase protein [Shigella phage Sf24] | Shigella phage Sf24 | 1E-116 | 99.39% | YP_009619098.1 |
| ORF96 | 66643 | 65825 | - | tail sheath stabilizer and completion protein | Shigella phage SHBML-50-1 | 0 | 100.00% | YP_009288528.1 |
| ORF97 | 67455 | 66685 | - | Phage neck protein | Enterobacteria phage RB51 | 0 | 100.00% | YP_002854119.1 |
| ORF98 | 68386 | 67457 | - | Phage neck protein | Escherichia phage vB_vPM_PD112 | 0 | 100.00% | YP_010073102.1 |
| ORF99 | 69876 | 68419 | - | Phage fibritin (wac) protein | Escherichia phage vB_vPM_PD112 | 0 | 99.79% | YP_010073101.1 |
| ORF100 | 71442 | 69886 | - | Phage straight tail fiber (short tail fiber) | Escherichia phage vB_vPM_PD112 | 0 | 100.00% | YP_010073100.1 |
| ORF101 | 72098 | 71439 | - | baseplate wedge subunit and tail pin | Escherichia phage vB_EcoM_IME537 | 3E-160 | 100.00% | YP_010071201.1 |
| ORF102 | 73903 | 72098 | - | hypothetical protein | Escherichia phage vB_EcoM_IME537 | 0 | 99.83% | YP_010071200.1 |
| ORF103 | 74769 | 73903 | - | Phage baseplate wedge tail fiber connector  (T4-like gp9) | Escherichia phage ECO4 | 0 | 100.00% | YP_010068391.1 |
| ORF104 | 75838 | 74834 | - | baseplate wedge subunit [Escherichia phage ECO4] | Escherichia phage ECO4 | 0 | 99.70% | YP_010068390.1 |
| ORF105 | 78929 | 75831 | - | Phage baseplate wedge initiator (T4-like gp7) | Enterobacteria phage RB27 | 0 | 99.71% | YP_009102358.1 |
| ORF106 | 80908 | 78926 | - | baseplate wedge protein [Escherichia phage N2] | Escherichia phage N2 | 0 | 99.85% | UTQ79472.1 |
| ORF107 | 81210 | 80917 | - | PAAR domain-containing protein  [Escherichia phage vB_EcoM-G28] | Escherichia phage vB_EcoM-G28 | 6E-64 | 98.97% | YP_010069712.1 |
| ORF108 | 81705 | 81211 | - | Phage protein | Escherichia phage W143 | 5E-103 | 100.00% | QWV60496.1 |
| ORF109 | 82606 | 81740 | - | hypothetical protein KMC37_gp252  [Yersinia phage vB_YepM_ZN18] | Yersinia phage vB_YepM_ZN18 | 0 | 99.31% | YP_010077651.1 |
| ORF110 | 84355 | 82625 | - | Phage baseplate hub structural protein /  Phage lysozyme R(EC 3.2.1.17) | Shigella phage vB_SsoM_113 | 0 | 99.65% | CAA7537964.1 |
| ORF111 | 84929 | 84339 | - | baseplate wedge protein 53  [Citrobacter phage PhiZZ6] | Citrobacter phage PhiZZ6 | 1E-141 | 99.49% | YP_010065518.1 |
| ORF112 | 84977 | 85429 | + | head completion protein [Escherichia phage MLF4] | Escherichia phage MLF4 | 2E-105 | 99.33% | YP_010099768.1 |
| ORF113 | 85429 | 86256 | + | DNA end protector protein [Yersinia phage fPS-2] | Yersinia phage fPS-2 | 0 | 99.64% | YP_010077029.1 |
| ORF114 | 86253 | 86909 | + | hypothetical protein G2133_00156  [Escherichia phage vB_EcoM_G2133] | Escherichia phage vB_EcoM_G2133 | 4E-158 | 98.63% | QBO60887.1 |
| ORF115 | 87016 | 87546 | + | Phage tail completion protein | Escherichia virus T4 | 2E-126 | 100.00% | AAA50419.1 |
| ORF116 | 87596 | 88309 | + | deoxynucleoside monophosphate kinase | Escherichia phage slur14 | 3E-174 | 99.16% | YP_009180646.1 |
| ORF117 | 88309 | 88551 | + | tail fiber assembly protein  [Escherichia phage vB_EcoM_Ozark] | Escherichia phage vB_EcoM_Ozark | 1E-45 | 98.75% | YP_010072708.1 |
| ORF118 | 88551 | 89006 | + | hypothetical protein KNT57_gp103  [Shigella phage KNP5] | Shigella phage KNP5 | 4E-107 | 99.34% | YP_010278270.1 |
| ORF119 | 89079 | 89366 | + | Phage protein | Escherichia phage HY01 | 1E-48 | 100.00% | YP_009148583.1 |
| ORF120 | 89443 | 89628 | + | hypothetical protein KMC13_gp255  [Escherichia phage vB_EcoM_IME537] | Escherichia phage vB_EcoM_IME537 | 1E-23 | 98.36% | YP_010071183.1 |
| ORF121 | 89630 | 90004 | + | Phage protein | Escherichia phage vB_EcoM_IME537 | 1E-85 | 98.39% | YP_010071182.1 |
| ORF122 | 90007 | 90294 | + | Phage protein | Citrobacter phage PhiZZ23 | 3E-63 | 100.00% | YP_010065783.1 |
| ORF123 | 91005 | 91670 | + | GIY-YIG nuclease family protein  [Escherichia phage PP01] | Escherichia phage PP01 | 2E-144 | 98.64% | YP_010073506.1 |
| ORF124 | 92142 | 92615 | + | hypothetical protein | Escherichia phage vB_vPM_PD112 | 8E-102 | 100.00% | YP_010073077.1 |
| ORF125 | 92856 | 93119 | + | Phage protein | Escherichia phage JLBYU22 | 7E-58 | 100.00% | UGO56668.1 |
| ORF126 | 93175 | 93705 | + | Phage protein | Salmonella phage pSe_SNUABM_01 | 8E-125 | 99.43% | YP_010075398.1 |
| ORF127 | 93749 | 94342 | + | hypothetical protein KMB91_gp085  [Citrobacter phage vB_CroM_CrRp10] | Citrobacter phage vB_CroM_CrRp10 | 1E-119 | 99.49% | YP_010065191.1 |
| ORF128 | 94384 | 94998 | + | Phage protein | Escherichia phage F2 | 2E-147 | 100.00% | YP_010069010.1 |
| ORF129 | 94967 | 95359 | + | hypothetical protein | Escherichia phage vB_EcoM_IME537 | 9E-78 | 100.00% | YP_010071172.1 |
| ORF130 | 95341 | 95703 | + | Phage protein | Escherichia phage UFV-AREG1 | 1E-60 | 99.17% | YP_009281465.1 |
| ORF131 | 95700 | 96140 | + | Nudix hydrolase, phage-associated | Escherichia phage vB_EcoM_IME537 | 2E-106 | 100.00% | YP_010071169.1 |
| ORF132 | 96177 | 96671 | + | baseplate hub + tail lysozyme  [Escherichia phage vB_EcoM_FT] | Escherichia phage vB_EcoM_FT | 1E-117 | 99.39% | QLF81126.1 |
| ORF133 | 96749 | 97222 | + | hypothetical protein ACQ54_gp115  [Escherichia phage HY01] | Escherichia phage HY01 | 7E-110 | 99.36% | YP_009148566.1 |
| ORF134 | 97364 | 97903 | + | Phage protein | Yersinia phage PYPS2T | 3E-129 | 98.32% | YP_010077386.1 |
| ORF135 | 97900 | 98229 | + | hypothetical protein AR1_124  [Escherichia phage AR1] | Escherichia phage AR1 | 9E-75 | 100.00% | YP_009167935.1 |
| ORF136 | 98236 | 98598 | + | Autonomous glycyl radical cofactor | Enterobacteria phage RB51 | 3E-82 | 100.00% | YP_002854079.1 |
| ORF137 | 98598 | 98819 | + | Phage protein | Shigella phage Shfl2 | 2E-44 | 98.63% | YP_004415016.1 |
| ORF138 | 98812 | 99078 | + | hypothetical protein | Enterobacteria phage vB_EcoM_IME340 | 1E-57 | 100.00% | YP_010066534.1 |
| ORF139 | 99078 | 99356 | + | Phage protein | Shigella phage Shfl2 | 3E-60 | 100.00% | YP_004415014.1 |
| ORF140 | 99416 | 99877 | + | site-specific RNase [Escherichia phage T2] | Escherichia phage T2 | 2E-109 | 99.35% | YP_010073768.1 |
| ORF141 | 99885 | 100430 | + | hypothetical protein | Escherichia phage vB_EcoM_IME537 | 1E-129 | 100.00% | YP_010071159.1 |
| ORF142 | 100423 | 100770 | + | Valyl-tRNA synthetase | Escherichia phage vB_EcoM_IME537 | 6E-78 | 100.00% | YP_010071158.1 |
| ORF143 | 100767 | 101234 | + | macro domain-containing protein [Shigella phage SHBML-50-1] | Shigella phage SHBML-50-1 | 7E-111 | 99.35% | YP_009288481.1 |
| ORF144 | 101231 | 101443 | + | Phage protein | Escherichia phage vB_EcoM_IME537 | 3E-45 | 100.00% | YP_010071156.1 |
| ORF145 | 101440 | 101646 | + | Phage protein | Shigella phage vB_SboM_Phaginator | 2E-43 | 100.00% | UGO46783.1 |
| ORF146 | 101643 | 101816 | + | Phage protein | Escherichia phage vB_EcoM_IME537 | 2E-31 | 100.00% | YP_010071154.1 |
| ORF147 | 101813 | 101998 | + | Phage protein | Escherichia phage FelixPlatter | 1E-35 | 100.00% | QXV78660.1 |
| ORF148 | 102008 | 102589 | + | thymidine kinase [Escherichia phage wV7] | Escherichia phage wV7 | 5E-141 | 99.48% | YP_007004852.1 |
| ORF149 | 102633 | 102845 | + | hypothetical protein BN81_113  [Yersinia phage phiD1] | Yersinia phage phiD1 | 3E-42 | 98.57% | YP_009149352.1 |
| ORF150 | 102858 | 103151 | + | rI membrane protein [Enterobacteria phage RB51] | Enterobacteria phage RB51 | 2E-53 | 98.97% | YP_002854065.1 |
| ORF151 | 103148 | 103534 | + | Phage protein | Escherichia phage wV7 | 1E-87 | 100.00% | YP_007004849.1 |
| ORF152 | 103630 | 103818 | + | hypothetical protein KMC13_gp220  [Escherichia phage vB_EcoM_IME537] | Escherichia phage vB_EcoM_IME537 | 1E-36 | 100.00% | YP_010071148.1 |
| ORF153 | 103818 | 104000 | + | Phage protein | Escherichia phage vB_EcoM_IME537 | 2E-32 | 100.00% | YP_010071147.1 |
| ORF154 | 104003 | 104197 | + | Phage protein | Escherichia phage vB_EcoM_DalCa | 1E-27 | 98.44% | YP_010067522.1 |
| ORF155 | 104187 | 104360 | + | Phage protein | Escherichia phage vB_EcoM_IME537 | 3E-33 | 100.00% | YP_010071145.1 |
| ORF156 | 104525 | 104710 | + | hypothetical protein KMB91_gp054  [Citrobacter phage vB_CroM_CrRp10] | Citrobacter phage vB_CroM_CrRp10 | 1E-34 | 96.72% | YP_010065160.1 |
| ORF157 | 104712 | 104978 | + | hypothetical protein | Escherichia phage vB_EcoM_IME537 | 7E-59 | 100.00% | YP_010071143.1 |
| ORF158 | 104980 | 105522 | + | Phage protein | Escherichia phage W143 | 8E-123 | 94.44% | QWV60444.1 |
| ORF159 | 105529 | 106050 | + | Phage protein | Phage NBEco003 | 2E-121 | 98.27% | YP_010106147.1 |
| ORF160 | 106053 | 106391 | + | Phage protein | Escherichia phage vB_EcoM_IME537 | 3E-52 | 97.96% | YP_010071140.1 |
| ORF161 | 106513 | 107523 | + | Thioredoxin, phage-associated | Escherichia phage vB_vPM_PD112 | 0 | 100.00% | YP_010073039.1 |
| ORF162 | 107641 | 107790 | + | hypothetical protein | Escherichia phage vB_EcoM_IME537 | 9E-26 | 100.00% | YP_010071138.1 |
| ORF163 | 107787 | 108050 | + | hypothetical protein | Escherichia phage ECML-134 | 8E-58 | 100.00% | YP_009102568.1 |
| ORF164 | 108120 | 109088 | + | Thioredoxin, phage-associated | Escherichia phage slur14 | 0 | 100.00% | YP_009180865.1 |
| ORF165 | 109191 | 109493 | + | Thioredoxin, phage-associated | Escherichia phage vB_EcoM_IME537 | 5E-68 | 100.00% | YP_010071134.1 |
| ORF166 | 109554 | 110081 | + | Thioredoxin, phage-associated | Escherichia phage vB_EcoM_IME537 | 2E-111 | 100.00% | YP_010071133.1 |
| ORF167 | 110137 | 110544 | + | Thioredoxin, phage-associated | Escherichia phage HY01 | 9E-79 | 100.00% | YP_009148536.1 |
| ORF168 | 110552 | 111442 | + | Thioredoxin, phage-associated | Escherichia phage EcNP1 | 0 | 98.99% | YP_010067999.1 |
| ORF169 | 111451 | 112464 | + | Thioredoxin, phage-associated | Escherichia phage vB_vPM_PD112 | 0 | 100.00% | YP_010073031.1 |
| ORF170 | 112492 | 113448 | + | Thioredoxin, phage-associated | Escherichia phage vB_vPM_PD112 | 0 | 100.00% | YP_010073030.1 |
| ORF171 | 113445 | 113762 | + | Thioredoxin, phage-associated | Escherichia phage vB_vPM_PD112 | 6E-72 | 100.00% | YP_010073029.1 |
| ORF172 | 113749 | 113922 | + | Thioredoxin, phage-associated | Escherichia phage vB_EcoM_IME537 | 3E-33 | 100.00% | YP_010071126.1 |
| ORF173 | 113991 | 114254 | + | putative glutaredoxin  [Escherichia phage vB_EcoM_G10400] | Escherichia phage vB_EcoM_G10400 | 9E-58 | 98.85% | QBO63601.1 |
| ORF174 | 114430 | 114639 | + | hypothetical protein | Escherichia phage vB_EcoM_IME537 | 1E-40 | 98.55% | YP_010071123.1 |
| ORF175 | 114762 | 115208 | + | Pin protease inhibitor | Escherichia phage vB_vPM_PD112 | 6E-89 | 100.00% | YP_010073023.1 |
| ORF176 | 115208 | 115384 | + | Phage protein | Escherichia phage vB_EcoM_IME537 | 7E-35 | 100.00% | YP_010071120.1 |
| ORF177 | 115427 | 115900 | + | endonuclease domain-containing protein  [Escherichia phage HP3] | Escherichia phage HP3 | 3E-111 | 98.73% | YP_010228878.1 |
| ORF178 | 115897 | 116442 | + | Ribonucleotide reductase of class III (anaerobic), large  subunit (EC 1.17.4.2) | Proteus phage Isf-Pm2 | 2E-132 | 100.00% | UNI72817.1 |
| ORF179 | 116683 | 117453 | + | Phage-associated homing endonuclease | Escherichia phage F2 | 0 | 100.00% | YP_010068795.1 |
| ORF180 | 117428 | 118759 | + | Ribonucleotide reductase of class III (anaerobic), large  subunit (EC 1.17.4.2) | Escherichia phage HY03 | 0 | 99.77% | YP_009284113.1 |
| ORF181 | 118752 | 119582 | + | Phage-associated homing endonuclease | Escherichia phage EC121 | 0 |  | YP_010067764.1 |
| ORF182 | 119579 | 120049 | + | Ribonucleotide reductase of class III (anaerobic),  activating protein (EC 1.97.1.4) | Escherichia phage vB_EcoM_SYGD1 | 6E-111 | 99.36% | QUD16271.1 |
| ORF183 | 120042 | 120155 | + | Phage protein | Enterobacteria phage vB_EcoM_IME340 | 2E-17 | 100% | YP_010066576.1 |
| ORF184 | 120164 | 120376 | + | hypothetical protein BI058_gp077  [Shigella phage SHBML-50-1] | Shigella phage SHBML-50-1 | 2E-29 | 98.57% | YP_009288443.1 |
| ORF185 | 120379 | 120687 | + | NrdH glutaredoxin [Escherichia phage ime09] | Escherichia phage ime09 | 3E-68 | 99.02% | YP_007004456.1 |
| ORF186 | 120847 | 121029 | + | Phage protein | Escherichia virus RB14 | 1E-34 | 100% | YP_002854407.1 |
| ORF187 | 121022 | 121315 | + | Phage protein | Shigella phage vB_SboM_Phaginator | 9E-63 | 100% | UGO46745.1 |
| ORF188 | 121323 | 121454 | + | Phage protein | Escherichia phage vB_EcoM_IME537 | 3E-23 | 100% | YP_010071109.1 |
| ORF189 | 121455 | 121655 | + | Phage protein | Shigella phage Shfl2 | 2E-41 | 100% | YP_004414969.1 |
| ORF190 | 121708 | 122034 | + | Phage protein | Shigella phage Shfl2 | 4E-72 | 100% | YP_004414968.1 |
| ORF191 | 122037 | 122252 | + | Phage protein | Enterobacteria phage vB_EcoM_IME340 | 1E-42 | 100% | YP_010066585.1 |
| ORF192 | 122249 | 122518 | + | Phage protein | Escherichia phage vB_vPM_PD112 | 5E-59 | 100% | YP_010073007.1 |
| ORF193 | 122597 | 123154 | + | T4-like phage RNA polymerase sigma factor for late  transcription | Escherichia phage vB_EcoM_112 | 2E-135 | 100% | YP_009030673.1 |
| ORF194 | 123168 | 123356 | + | Phage protein (ACLAME 855) | Shigella phage Shfl2 | 9E-36 | 100% | YP_004414964.1 |
| ORF195 | 123358 | 123675 | + | putative endonuclease  [Shigella phage vB_SboM_Phaginator] | Shigella phage vB_SboM_Phaginator | 0 | 99.71% | UGO46733.1 |
| ORF196 | 123851 | 124024 | + | Phage protein | Escherichia phage ime09 | 4E-34 | 100% | YP_007004444.1 |
| ORF197 | 124091 | 125293 | + | a-gt alpha glucosyl transferase | Shigella phage Shfl2 | 0 | 100% | YP_004414960.1 |
| ORF198 | 125470 | 126489 | + | Phage protein [Yersinia phage fPS-2] | Yersinia phage fPS-2 | 1E-55 | 97.70% | YP_010076942.1 |
| ORF199 | 126486 | 126749 | + | gp46.1 hypothetical protein [Tequatrovirus RB14] | Tequatrovirus RB14 | 1E-40 | 98.53% | YP_002854391.1 |
| ORF200 | 126730 | 126936 | + | sliding clamp DNA polymerase accessory protein [Escherichia phage vb_EcoM_bov9_1] | Escherichia phage vb_EcoM_bov9_1 | 2E-164 | 99.56% | QNR52799.1 |
| ORF201 | 126933 | 128615 | + | Phage recombination-related endonuclease Gp46 | Escherichia phage vB_vPM_PD112 | 0 | 100% | YP_010072997.1 |
| ORF202 | 128671 | 128859 | + | Phage protein | Escherichia phage vB_EcoM_ACG-C40 | 8E-38 | 100% | YP_006986607.1 |
| ORF203 | 128869 | 129258 | + | RNA polymerase binding protein | Escherichia phage vB_vPM_PD112 | 2E-91 | 100% | YP_010072995.1 |
| ORF204 | 129314 | 130000 | + | DNA polymerase clamp loader subunit  [Escherichia phage vB_EcoM-CHD94UKE2] | Escherichia phage vB_EcoM-CHD94UKE2 | 2E-124 | 100.00% | QZI80988.1 |
| ORF205 | 130052 | 131011 | + | Replication factor C small subunit / Phage DNA polymerase clamp loader subunit | Escherichia phage vB_EcoM_IME537 | 0 | 100% | YP_010071091.1 |
| ORF206 | 131013 | 131576 | + | translational repressor RegA [Shigella phage pSs-1] | Shigella phage pSs-1 | 1E-84 | 99.18% | YP_009110868.1 |
| ORF207 | 131578 | 131946 | + | hypothetical protein KMC13_gp159 [Escherichia phage vB_EcoM_IME537] | Escherichia phage vB_EcoM_IME537 | 5E-117 | 99.39% | YP_010071087.1 |
| ORF208 | 132025 | 134721 | + | DNA polymerase | Escherichia phage vB_vPM_PD112 | 0 | 100% | YP_010072990.1 |
| ORF209 | 134793 | 135284 | + | hypothetical protein KMC13_gp159  [Escherichia phage vB_EcoM_IME537] | Escherichia phage vB_EcoM_IME537 | 5E-117 | 99.39% | YP_010071087.1 |
| ORF210 | 135292 | 135543 | + | superinfection immunity protein  [Citrobacter phage PhiZZ23] | Citrobacter phage PhiZZ23 | 2E-49 | 98.80% | YP_010065687.1 |
| ORF211 | 135697 | 136437 | + | hypothetical protein | Escherichia phage vB_vPM_PD112 | 0 | 100% | YP_010072987.1 |
| ORF212 | 136434 | 137276 | + | glucosyl transferase | Escherichia phage vB_vPM_PD112 | 0 | 100% | YP_010072986.1 |
| ORF213 | 137281 | 137946 | + | Phage-associated homing endonuclease | Escherichia phage vB_vPM_PD112 | 8E-151 | 100% | YP_010072985.1 |
| ORF214 | 137955 | 139130 | + | Phage recombination protein | Escherichia phage vB_vPM_PD112 | 0 | 100% | YP_010072984.1 |
| ORF215 | 139123 | 139467 | + | Phage capsid and scaffold | Citrobacter phage PhiZZ23 | 1E-59 | 100% | YP_010065683.1 |
| ORF216 | 139477 | 140904 | + | Phage DNA primase/helicase | Escherichia phage vB_EcoM_ACG-C40 | 0 | 99.79% | YP_006986590.1 |
| ORF217 | 140963 | 141145 | + | putative discriminator of mRNA degradation [Shigella phage vB_SboM_Phaginator] | Shigella phage vB_SboM_Phaginator | 9E-35 | 98.33% | UGO46713.1 |
| ORF218 | 141147 | 141368 | + | Phage protein | Escherichia phage vB_EcoM-G28 | 9E-46 | 100% | YP_010069603.1 |
| ORF219 | 141453 | 141707 | + | hypothetical protein | Shigella phage Shfl2 | 6E-45 | 100% | YP_004414938.1 |
| ORF220 | 141765 | 142058 | + | hypothetical protein | Escherichia phage vB_vPM_PD112 | 1E-55 | 100% | YP_010072979.1 |
| ORF221 | 142071 | 142427 | + | Phage protein | Enterobacteria phage RB27 | 6E-80 | 100% | YP_009102240.1 |
| ORF222 | 142429 | 142593 | + | hypothetical protein | Escherichia phage vB_EcoM_Shinka | 7E-21 | 100% | QXV73253.1 |
| ORF223 | 142596 | 143624 | + | DNA primase (EC 2.7.7.-) / DNA helicase (EC 3.6.1.-),  phage-associated | Escherichia phage vB_vPM_PD112 | 0 | 100% | YP_010072976.1 |
| ORF224 | 143821 | 143621 | - | Phage protein | Escherichia phage vB_vPM_PD112 | 3E-37 | 100% | YP_010072975.1 |
| ORF225 | 143930 | 144709 | + | DNA adenine methyltransferase, phage-associated | Escherichia phage vB_vPM_PD112 | 3E-173 | 100% | YP_010072974.1 |
| ORF226 | 144773 | 145288 | + | dCTP pyrophosphatase (EC 3.6.1.12), phage-associated  (ACLAME 965) | Escherichia phage vB_vPM_PD112 | 4E-124 | 100% | YP_010072973.1 |
| ORF227 | 145288 | 145488 | + | Phage protein | Escherichia phage vB_vPM_PD112 | 5E-39 | 100% | YP_010072972.1 |
| ORF228 | 145485 | 145658 | + | hypothetical protein | Escherichia phage vB_EcoM_DalCa | 1E-32 | 100% | YP_010067448.1 |
| ORF229 | 145698 | 145934 | + | hypothetical protein | Escherichia phage vB_vPM_PD112 | 3E-50 | 100% | YP_010072970.1 |
| ORF230 | 146033 | 146245 | + | Phage protein | Enterobacteria phage GiZh | 4E-45 | 100% | YP_010066243.1 |
| ORF231 | 146245 | 146577 | + | Phage protein | Shigella phage CT01 | 0.00003 | 100% | UDY80419.1 |
| ORF232 | 146586 | 147071 | + | Phage protein | Escherichia phage BF15 | 1E-108 | 98.14% | QXN75730.1 |
| ORF233 | 147246 | 147410 | + | putative 6.2 kDa protein [Salmonella phage Lv5cm] | Salmonella phage Lv5cm | 2E-29 | 98.15% | QVW08833.1 |
| ORF234 | 147403 | 147873 | + | Molybdenum ABC transporter, periplasmic molybdenum -binding protein ModA (TC 3.A.1.8.1) | Escherichia phage vB_vPM_PD112 | 4E-108 | 98.08% | YP_010072964.1 |
| ORF235 | 147882 | 148064 | + | Molybdenum ABC transporter, periplasmic molybdenum -binding protein ModA (TC 3.A.1.8.1) | Enterobacteria phage RB51 | 4E-36 | 100% | YP_002853978.1 |
| ORF236 | 148132 | 148755 | + | NAD--protein ADP-ribosyltransferase modA  (EC 2.4.2.-) | Salmonella phage Lv5cm | 3E-151 | 100% | QVW08830.1 |
| ORF237 | 148752 | 149354 | + | NAD--protein ADP-ribosyltransferase modA  (EC 2.4.2.-) | Escherichia phage HP3 | 6E-147 | 99.50% | YP_010228941.1 |
| ORF238 | 149471 | 150217 | + | putative anti-sigma factor | Escherichia phage vB_EcoM_IME537 | 3E-178 | 100% | YP_010071056.1 |
| ORF239 | 150219 | 150530 | + | putative 12.1 kDa protein [Escherichia phage W143] | Escherichia phage W143 | 1E-67 | 99.03% | QWV60366.1 |
| ORF240 | 150527 | 151846 | + | DNA helicase (EC 3.6.1.-), phage-associated | Shigella phage Shfl2 | 0 | 100% | YP_004414916.1 |
| ORF241 | 151868 | 152113 | + | Phage exonuclease | Escherichia phage vB_vPM_PD112 | 4E-49 | 100% | YP_010072957.1 |
| ORF242 | 152106 | 152348 | + | Phage exonuclease | Escherichia phage vB_EcoM_IME537 | 1E-49 | 100% | YP_010071052.1 |
| ORF243 | 152348 | 153031 | + | exonuclease | Escherichia phage HY01 | 1E-167 | 100% | YP_009148464.1 |
| ORF244 | 153095 | 153595 | + | Transcriptional regulator | Escherichia phage vB_vPM_PD112 | 2E-120 | 100% | YP_010072954.1 |
| ORF245 | 153598 | 154143 | + | Transcriptional regulator | Escherichia phage vB_EcoM_IME537 | 1E-129 | 100% | YP_010071049.1 |
| ORF246 | 154220 | 154717 | + | Transcriptional regulator | Escherichia phage vB_vPM_PD112 | 3E-116 | 99.39% | YP_010072952.1 |
| ORF247 | 154889 | 155104 | + | Phage cef modifier of suppressor tRNAs (ACLAME 1240) | Escherichia phage HY01 | 8E-45 | 100% | YP_009148460.1 |
| ORF248 | 155104 | 155517 | + | Phage protein | Escherichia phage vB_vPM_PD112 | 8E-97 | 100% | YP_010072950.1 |
| ORF249 | 155520 | 155696 | + | zinc ribbon domain-containing protein  [Escherichia phage vB_EcoM_DalCa] | Escherichia phage vB_EcoM_DalCa | 2E-34 | 98.28% | YP_010067425.1 |
| ORF250 | 155699 | 155959 | + | Phage protein | Escherichia phage vB_EcoM_G4498 | 2E-56 | 100% | YP_010070103.1 |
| ORF251 | 156029 | 157579 | + | Phage DNA topoisomerase large subunit (EC 5.99.1.3) | Escherichia phage vB_vPM_PD112 | 0 | 100% | YP_010072948.1 |
| ORF252 | 157557 | 158375 | + | Phage protein | Escherichia phage vB_EcoM_112 | 0 | 100% | YP_009030888.1 |
| ORF253 | 158356 | 158496 | + | Phage DNA topoisomerase large subunit (EC 5.99.1.3) | Escherichia phage vB_EcoM_G50 | 2E-23 | 100% | YP_010069836.1 |
| ORF254 | 158751 | 158888 | + | Phage DNA topoisomerase large subunit (EC 5.99.1.3) | Escherichia phage vB_EcoM_G50 | 8E-24 | 100% | YP_010069835.1 |
| ORF255 | 158943 | 159146 | + | Phage rIIA lysis inhibitor | Escherichia phage vB_EcoM_IME537 | 1E-39 | 100% | YP_010071042.1 |
| ORF256 | 159157 | 161334 | + | Phage rIIA lysis inhibitor | Escherichia phage vB_vPM_PD112 | 0 | 99.72% | YP_010072943.1 |
| ORF257 | 161346 | 162284 | + | 1 | Escherichia phage vB_EcoM_IME537 | 0 | 99.68% | YP_010071040.1 |
| ORF258 | 162317 | 162511 | + | Phage endonuclease | Enterobacteria phage RB51 | 3E-36 | 100% | YP_002854227.1 |
| ORF259 | 162550 | 162879 | + | Phage protein | Escherichia phage vB_EcoM_IME537 | 1E-57 | 100% | YP_010071038.1 |
| ORF260 | 162893 | 163369 | + | Phage endonuclease | Escherichia phage vB_EcoM_IME537 | 2E-115 | 100% | YP_010071037.1 |
| ORF261 | 163451 | 163657 | + | Phage protein | Escherichia virus RB14 | 8E-28 | 100% | YP_002854606.1 |
| ORF262 | 163663 | 163926 | + | Phage protein | Shigella phage vB_SboM_Phaginator | 1E-57 | 100% | UGO46937.1 |
| ORF263 | 164006 | 164104 | + | Phage protein | Escherichia phage wV7 | 7E-13 | 100% | YP_007005010.1 |
| ORF264 | 164170 | 164283 | + | Phage protein | Escherichia phage U115 | 4E-15 | 100% | UAV89232.1 |
| ORF265 | 164291 | 164488 | + | Phage protein | Escherichia phage vB_EcoM_IME537 | 3E-38 | 100% | YP_010071034.1 |
| ORF266 | 164604 | 164819 | + | Phage protein | Escherichia phage vB_vPM_PD112 | 5E-44 | 100% | YP_010072934.1 |
| ORF267 | 164880 | 165335 | + | naphthalene 1,2-dioxygenase  [Escherichia phage vB_EcoM_G50] | Escherichia phage vB_EcoM_G50 | 2E-108 | 99.34% | YP_010070090.1 |
| ORF268 | 165423 | 165578 | + | Acridine resistance | Escherichia virus T4 | 1E-26 | 100% | NP_049877.1 |
| ORF269 | 165716 | 167044 | + | DNA topoisomerase, phage-associated | Escherichia phage vB_EcoM-UFV13 | 0 | 100% | YP_009290524.1 |
| ORF270 | 167041 | 167190 | + | putative 4.8 kDa protein [Salmonella phage Lv5cm] | Salmonella phage Lv5cm | 0.021 | 97.96% | QVW09068.1 |
| ORF271 | 167317 | 167952 | + | putative activator of middle period transcription [Escherichia phage vB_EcoM_NBG2] | Escherichia phage vB_EcoM_NBG2 | 3E-150 | 99.53% | YP_010072271.1 |
