# Supplementary material for: Characterization of a lytic Escherichia coli phage CE1 and its potential use in therapy against avian pathogenic Escherichia coli infections
Source: Front Microbiol. 2023 Feb 16;14:1091442. doi: 10.3389/fmicb.2023.1091442 (PMC9978775; doi:10.3389/fmicb.2023.1091442)
Supplement: Supplementary file 3 [file Table_3.DOCX]

| Genome characteristics | Phage | | | | | | |
| --- | --- | --- | --- | --- | --- | --- | --- |
|  | CE1 | T4 | HY01 | JB75 | AR1 | SYGD1 | SV76 |
| Genome size (bp) | 167,955 | 168,903 | 166,977 | 167,208 | 167,435 | 171,255 | 163,826 |
| G+C% | 35.42 | 34.5 | 35.5 | 35 | 35.3 | 35.33 | 35.3 |
| Predicted ORFs | 271 | 289 | 258 | 277 | 281 | 271 | 287 |
| tRNAs | 8 | 8 | 9 | 0 | 10 | 8 | 9 |
| GenBank accession no. | ON229909 | AF158101 | KF925357 | MH355584 | AP011113 | [MW883059](https://www.ncbi.nlm.nih.gov/nucleotide/MW883059.1?report=genbank&log$=nucltop&blast_rank=21&RID=MM65VBZN013) | OM339528 |

Table S3 General genome features of phage CE1 and other closely related phages
