# Supplementary figures and images for: Characterization of a lytic Escherichia coli phage CE1 and its potential use in therapy against avian pathogenic Escherichia coli infections
Source: Front Microbiol. 2023 Feb 16;14:1091442. doi: 10.3389/fmicb.2023.1091442 (PMC9978775; doi:10.3389/fmicb.2023.1091442)

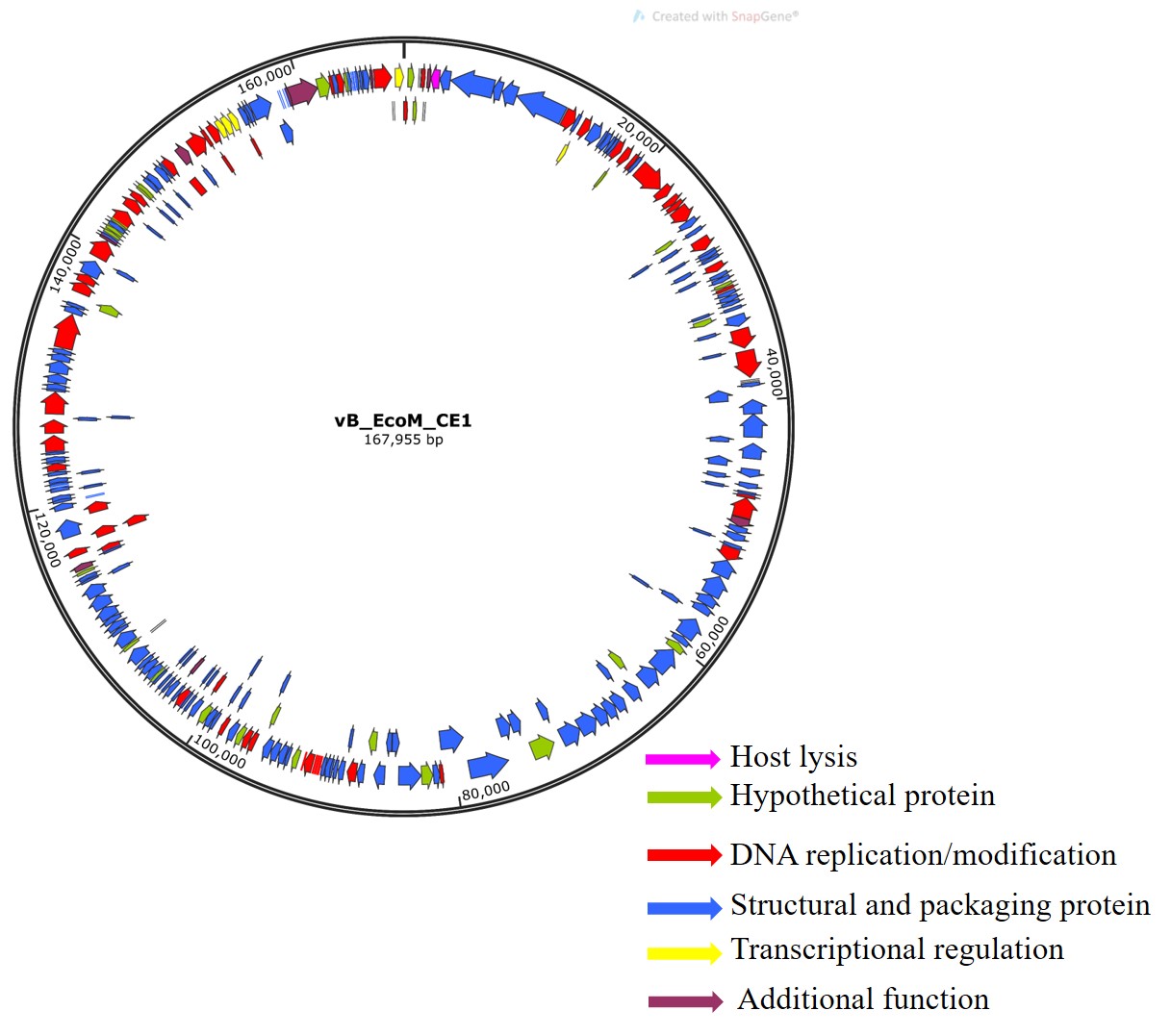

Supplement: Supplementary file 4 [file Image_1.JPEG]

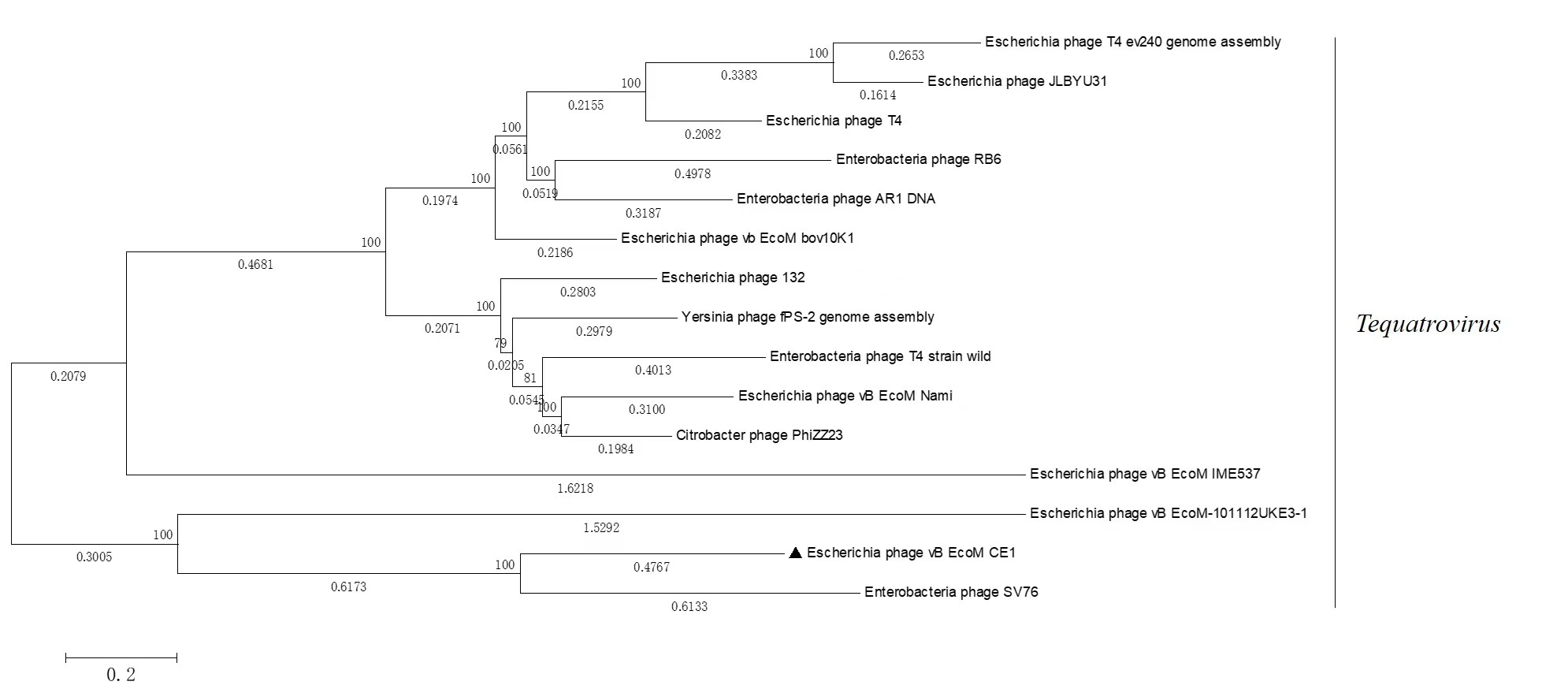

Supplement: Supplementary file 5 [file Image_2.JPEG]

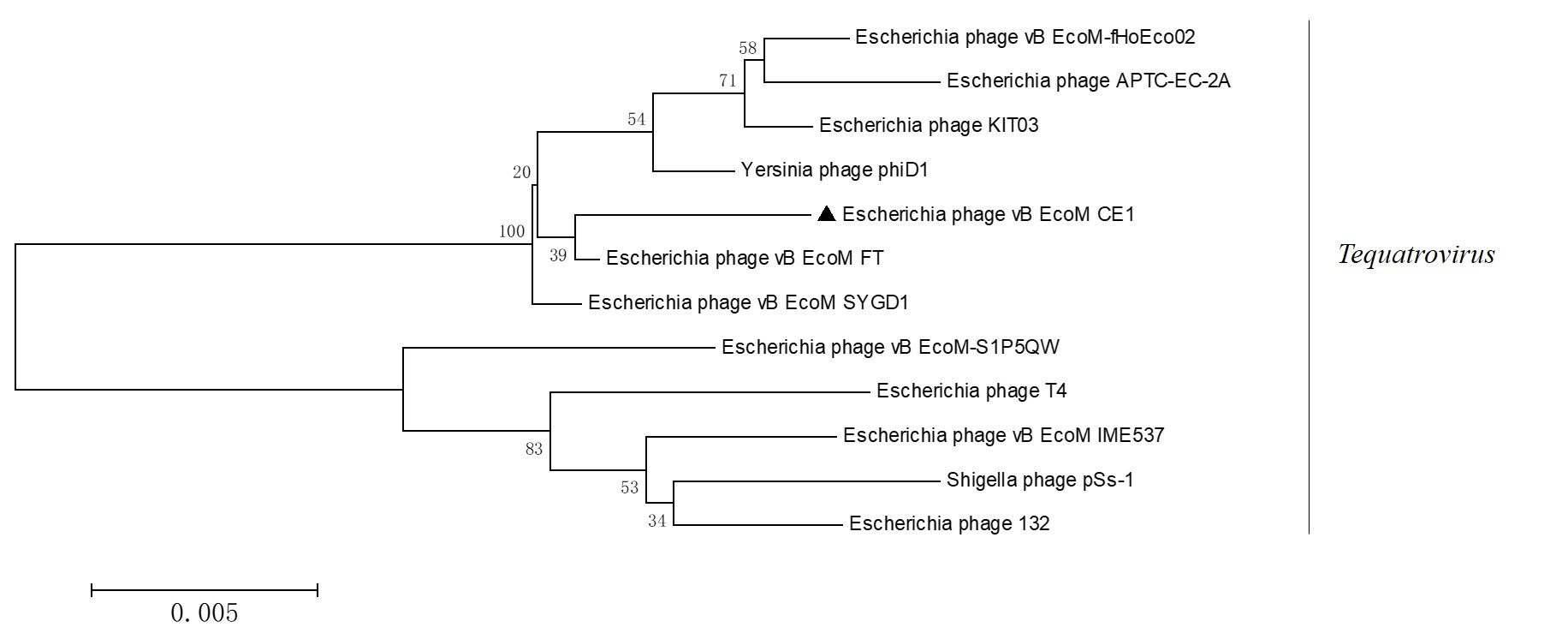

Supplement: Supplementary file 6 [file Image_3.JPEG]

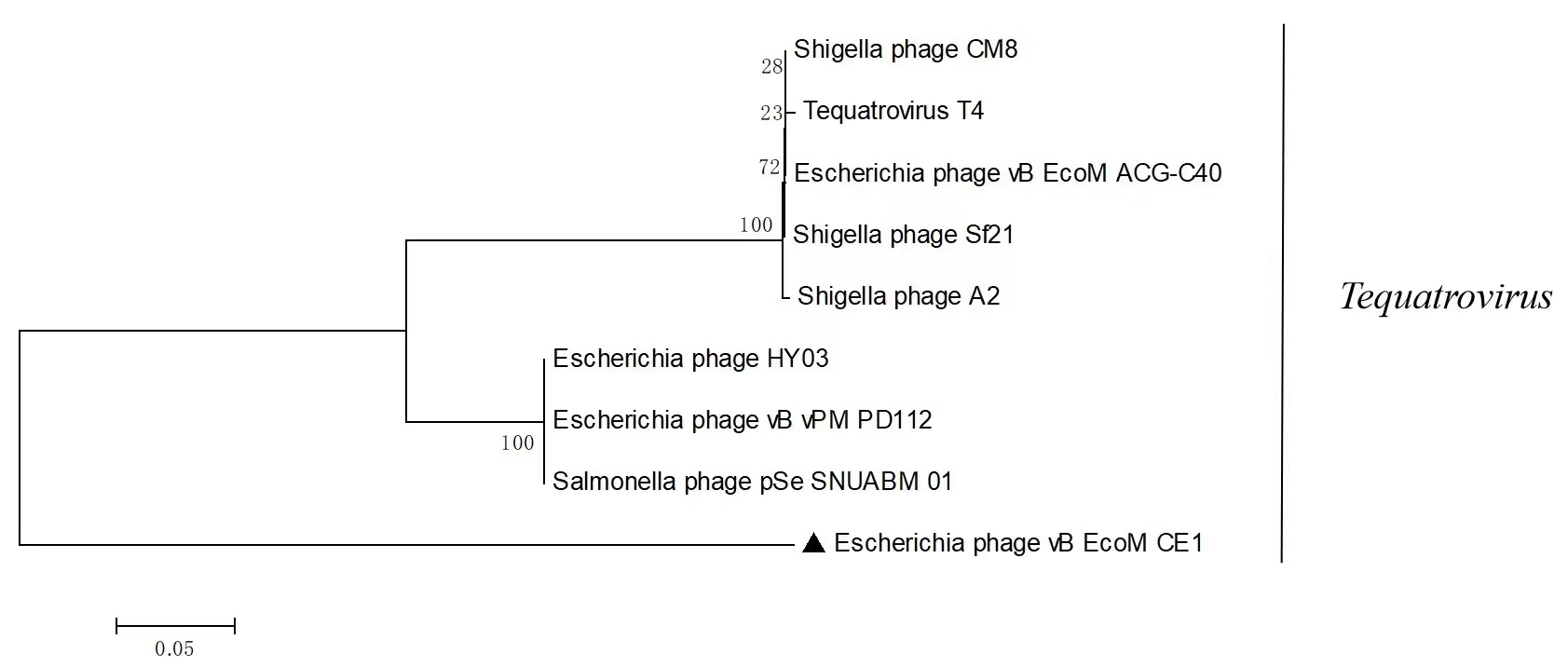

Supplement: Supplementary file 7 [file Image_4.JPEG]
